# Targeted RNA-Sequencing with Competitive Multiplex-PCR Amplicon Libraries

**DOI:** 10.1371/journal.pone.0079120

**Published:** 2013-11-13

**Authors:** Thomas M. Blomquist, Erin L. Crawford, Jennie L. Lovett, Jiyoun Yeo, Lauren M. Stanoszek, Albert Levin, Jia Li, Mei Lu, Leming Shi, Kenneth Muldrew, James C. Willey

**Affiliations:** 1 Department of Medicine, University of Toledo Health Sciences Campus, Toledo, Ohio, United States of America; 2 Department of Pathology, University of Toledo Health Sciences Campus, Toledo, Ohio, United States of America; 3 Department of Public Health Sciences, Henry Ford Health System, Detroit, Michigan, United States of America; 4 National Center for Toxicological Research, US Food and Drug Administration, Jefferson, Arkansas, United States of America; Boston University Medical Center, United States of America

## Abstract

Whole transcriptome RNA-sequencing is a powerful tool, but is costly and yields complex data sets that limit its utility in molecular diagnostic testing. A targeted quantitative RNA-sequencing method that is reproducible and reduces the number of sequencing reads required to measure transcripts over the full range of expression would be better suited to diagnostic testing. Toward this goal, we developed a competitive multiplex PCR-based amplicon sequencing library preparation method that a) targets only the sequences of interest and b) controls for inter-target variation in PCR amplification during library preparation by measuring each transcript native template relative to a known number of synthetic competitive template internal standard copies. To determine the utility of this method, we intentionally selected PCR conditions that would cause transcript amplification products (amplicons) to converge toward equimolar concentrations (normalization) during library preparation. We then tested whether this approach would enable accurate and reproducible quantification of each transcript across multiple library preparations, and at the same time reduce (through normalization) total sequencing reads required for quantification of transcript targets across a large range of expression. We demonstrate excellent reproducibility (R^2^ = 0.997) with 97% accuracy to detect 2-fold change using External RNA Controls Consortium (ERCC) reference materials; high inter-day, inter-site and inter-library concordance (R^2^ = 0.97–0.99) using FDA Sequencing Quality Control (SEQC) reference materials; and cross-platform concordance with both TaqMan qPCR (R^2^ = 0.96) and whole transcriptome RNA-sequencing following “traditional” library preparation using Illumina NGS kits (R^2^ = 0.94). Using this method, sequencing reads required to accurately quantify more than 100 targeted transcripts expressed over a 10^7^-fold range was reduced more than 10,000-fold, from 2.3×10^9^ to 1.4×10^5^ sequencing reads. These studies demonstrate that the competitive multiplex-PCR amplicon library preparation method presented here provides the quality control, reproducibility, and reduced sequencing reads necessary for development and implementation of targeted quantitative RNA-sequencing biomarkers in molecular diagnostic testing.

## Introduction

Next-generation sequencing (NGS) of RNA derived templates, or RNA-sequencing, is a powerful method with potential to rapidly advance discovery, development and implementation of transcript-based biomarkers in the clinical setting [1]. Whole transcriptome RNA-sequencing provides transcript abundance quantification along with detailed transcript structure information, including representation of alternative transcripts, polymorphisms, and mutations, including translocations. However, the cost and complexity of whole transcriptome RNA-sequencing data sets are barriers to use of this method in routine molecular diagnostic testing. For example, quantification of expression levels as low as 1 transcript/cell with adequate precision typically requires >10^8^ sequencing reads [2]. Recently developed targeted approaches reduce data complexity, and, to some extent, cost due to their focused nature [3]–[8]. However, targeted approaches reported thus far have limited clinical utility for at least two reasons:

1. Target enrichment steps, including bait hybridization-, capture and ligation- or polymerase chain reaction (PCR)-based strategies, for RNA-sequencing may be associated with inter-library variation in measurement of transcript expression [3]–[11]. Since these targeted methods for RNA-sequencing recently were developed the specific causes of inter-library variation in measurement have not yet been reported. However, it is reasonable to extrapolate from observations reported with similar techniques as to how variation may occur. As an example, for each of these approaches there may be inter-target variation in melting temperature of hybridization between native nucleic acid targets and enrichment templates. Further, disparity in annealing efficiency may be accentuated by inter-sample and inter-laboratory variation in conditions [12]. In particular, for multiplex PCR-based amplicon library preparation, sample overloading or excessive amplification cycles may cause different targets to plateau at different cycles depending on the level of expression, the amount of sample loaded, and the total number of cycles used. Array- or solution-based bait enrichment targeted sequencing libraries also are likely to be susceptible to potential overloading and signal saturation effects as is observed with the limited dynamic range in microarray expression measurements [2]. This can lead to inter-experimental and inter-target variation in signal compression. One way to avoid these effects is to routinely load less sample and/or use fewer amplification cycles for PCR based targeted sequencing approaches [8]. This concept is similar to the earliest semi-quantitative end-point PCR methods, when PCR was performed on serial dilutions of target templates in an effort to find a concentration that yields detectable signal yet is not at plateau phase for any of the targets being measured [12]–[14]. Historically, these steps can result in loss of signal for lowly expressed target transcripts and false negative reporting. Further, the relative abundance of the highest expressed transcript targets typically is unknown making it difficult to ensure prevention of early plateau in any given experiment. In addition, inter-sample variation in target-specific amplification inhibitors, commonly observed among clinical samples, may cause non-systematic analytical variation and contribute to false negative reporting [15]–[19]. These sources of non-systematic variation, if not adequately controlled, will confound inter-sample comparison of transcript abundance data required for clinical diagnostic testing or collection of data for submission to regulatory agencies obtained using current targeted quantitative RNA-sequencing approaches.
2. Since a goal of many targeted RNA-sequencing methods is to maintain the initial relative quantitative representation of targets, a large number of sequencing reads is still required to reproducibly quantify each of them [20]. Specifically, the large range in expression typically exhibited among targeted transcripts in a given sample imposes a need for over-sequencing of the highest expressed transcript target in order to accurately quantify the lowest [2]. In turn, this reduces the number of samples that can be evaluated during each sequencing run, and therefore increases direct sequencing costs per sample [21]. A targeted method which reduces the over-sequencing of highly expressed transcripts relative to lowly expressed ones yet maintains information regarding the original quantitative relationship between targets is needed for targeted quantitative RNA-sequencing to be cost-effective in the clinical setting.

**Figure 1:**
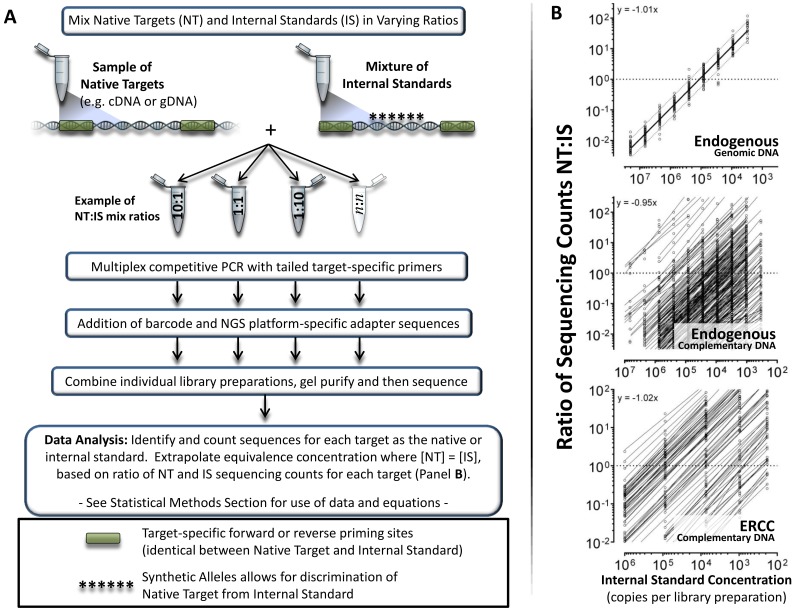
Competitive amplicon library preparation workflow and data analysis overview. **a)** Competitive amplicon library preparation workflow. An internal standard (IS) is a single-stranded or double-stranded DNA template that a) is homologous to a specific native target (NT) at the primer sequences (green shaded regions) and therefore competes for amplification with the NT but b) contains one or more base substitutions (depicted by asterisks) internal to the primer sites and therefore is distinguishable from the NT during post-sequencing data analysis. The corresponding IS template for each native target is in a fixed relationship relative to the IS for the other genes in an internal standard mixture, and the concentration of each IS in the mixture is known. The native sample and competitive internal standard mixture are combined in varying ratios and processed according to the flow diagram (details available in Methods section). **b)** Linearity of titration between competitive IS relative to native targets in samples A-D (samples used are described in detail in Methods section). With competitive methodologies, it is important to demonstrate that as the abundance of competitive internal standard mixture changes relative to native material (x-axis), the ratio of measured native material to competitive internal standards changes in direct proportion (y-axis), and that the slope is near to 1.00. The proportional relationship among native targets in the original sample is preserved during amplification and sequencing because i) the competition between each NT and its respective IS enables calculation of the original concentration for each NT, and ii) the IS are in a fixed relationship relative to each other (**Figure 2** and **Animation S1**). Native targets for which values could not be measured across at least three dilution points are not shown. Upper panel: Linearity of cross titrating competitive Internal Standard Mixture with constant amount of genomic DNA (gDNA) for 123 targets (10 titration points). Dotted lines represent 95% prediction interval for NT:IS ratio values. Middle panel: Linearity of cross titrating competitive Internal Standard Mixture with constant amount of endogenous cDNA native targets from samples A–D (same targets as assessed in gDNA; upper panel) (12 titration points). Lower panel: Linearity of cross-titrating competitive Internal Standard Mixture with constant amount of 26 ERCC native targets from samples A–D (5 titration points). Each titration and assay measurement represents a single technical replicate.

**Figure 2:**
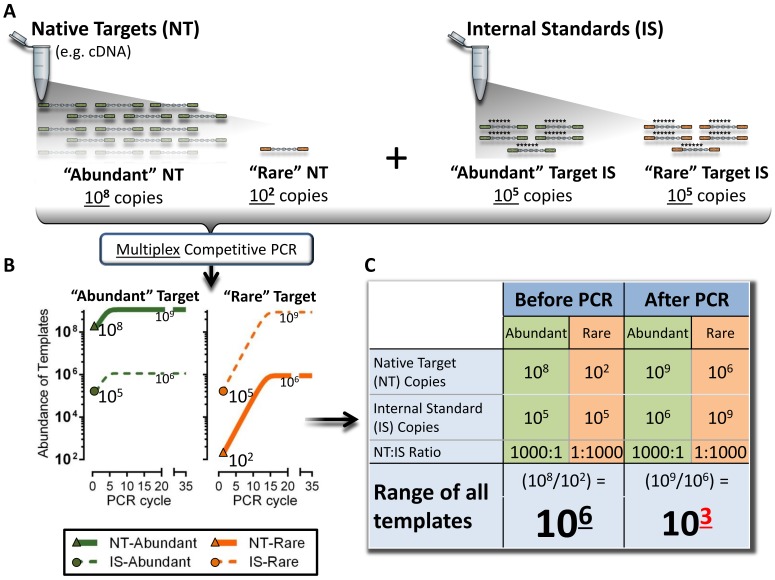
Schematic depiction of how competitive amplicon library preparation reduces oversampling. **a)** Depicted are two native targets (NT) within a hypothetical cDNA sample. One NT is in high abundance, 10^8^ copies (“Abundant” NT), while another is in low abundance, 10^2^ copies (“Rare” NT), representing a one million-fold difference in abundance between targets. This hypothetical cDNA sample is combined with a mixture of internal standards (IS) with a fixed relationship of concentrations at 10^5^ copies. **b)** Depicted is the competitive multiplex-PCR library preparation for panel A. The PCR amplification plots for both the “Abundant” and “Rare” NT are separated for purposes of clarity, but occur in the same reaction. During competitive multiplex-PCR, each NT competes equally with its respective competitive IS for dNTPs, polymerase and a limiting concentration of primers. Because the starting concentration of each target’s primer-pair is the same, each competitive reaction will plateau around the same end-point concentration (∼10^9^ copies). **c)** The equal competition between each NT and respective IS preserves the proportional relationship between NT in the original sample, allowing for measurement of native target abundance without signal compression (also see **Animation S1**). Yet, a 10^6^ fold range of templates is reduced to 10^3^ after competitive multiplex-PCR library preparation resulting in a 1,000-fold reduction in oversampling/sequencing of the high abundance target.

We hypothesized that a competitive approach to multiplex PCR-based amplicon library preparation would effectively address the quality-control and cost limitations associated with existing PCR-based targeted RNA-sequencing methods [5], [7]. With competitive amplicon library preparation, each native target (NT) in a sample (e.g. cDNA) is multiplex PCR-amplified in the presence of a known number of its respective competitive internal standard (IS) molecules within an IS mixture [13], [22] (**Figures 1** and **2**). Introduction of a competitive IS mixture into PCR-based reactions controls for inter-sample variation in the presence of interfering substances [16]–[19]. In addition, use of the same competitive IS mixture across PCR experiments and laboratories controls for multiple sources of analytical variation ensuring concordance among data sets [2], [15]–[17], [23]. After sequencing a library prepared with competitive multiplex PCR, the number of sequencing reads for each native target is measured relative to the number of reads observed for its respective competitive IS. We reasoned that this approach would enable highly reproducible quantitative targeted RNA-sequencing.

Introduction of a competitive IS mixture into PCR-based reactions also controls for inter-target variation in amplification efficiency due to limitation in reagent quality or quantity [16]–[19]. Specifically, because each target native template (NT) amplifies with the same efficiency as its respective competitive IS, the pair remains in a fixed proportion (NT:IS ratio) throughout amplicon library preparation (**Figures 1** **,** **2** and **Animation S1**). Since the NT:IS ratio remains fixed, the starting number of NT molecules can be determined by multiplying the NT:IS ratio at the end of PCR by the known number of IS molecules placed into the library preparation (**Figure 1**). Importantly, inter-target convergence (i.e. normalization between amplicons) from original representation is controlled because each target NT is measured relative to a known starting number of competitive IS copies (**Figure 2** **and Animation S1**). Taking advantage of this characteristic, we designed amplification conditions that promote convergence of inter-transcript native amplicon representation toward equimolar proportions at plateau phase (**Figure 2** with details in Methods section and **Animation S1**). Attaining this goal would be important because convergence of representation would reduce the need to over-sequence high abundance templates, enabling analysis of more samples per sequencing chip and thereby reducing direct sequencing costs. The cost savings potential with this approach would be substantial because inter-gene variation in transcript representation commonly ranges more than one million-fold [2].

In this study, we assessed the utility of the competitive multiplex PCR amplicon library preparation method to support reproducible and cost-effective transcript abundance measurement on the Ion Torrent NGS platform. To do this, we used External RNA Controls Consortium (ERCC) and FDA-sponsored Sequencing Quality Control (SEQC) project reference material RNA cross-titration pools (http://www.fda.gov/ScienceResearch/BioinformaticsTools/MicroarrayQualityControlProject/) [2], [24]. We prepared libraries using primers and competitive IS for 178 endogenous and synthetic cDNA targets expressed over a greater than 10^7^-fold range and subjected them to IonTorrent NGS. We then evaluated the method for: a) accuracy and reproducibility of nucleic acid abundance measurement on different days within individual test sites, between test sites, and between different preparations of libraries, b) inter-platform concordance with TaqMan qPCR (MAQC-I study) and traditional RNA-sequencing library preparation using Illumina NGS kits (SEQC study), as well as c) reduced number of sequencing reads required for quantification.

## Methods

### Competitive Amplicon Library Preparation Concept

A schematic of the workflow and data analysis for competitive amplicon library preparation is depicted in **Figures 1** and **2**, with a detailed description provided in the following Method sections, and animated schematic depicting the core concepts in **Animation S1**.

### Samples

#### Genomic DNA test and normalization material

Genomic DNA (gDNA) was extracted by FlexiGene DNA kit (Qiagen), quantified using commercially available genomic DNA qPCR reagents (Accugenomics, Wilmington, NC), and diluted to a stock concentration of 100,000 genomic copies per µL in dilute TE buffer (10 mM Tris-Cl, pH 7.4, 0.1 mM EDTA). Ethics statement: Peripheral whole blood (20 ml) was obtained by phlebotomy from subject ID 723 at the University of Toledo Medical Center (UTMC) under the University of Toledo Biomedical Institutional Review Board (IRB) approved and confirmed protocol number 105081. Both written and oral consent were obtained.

#### RNA reference materials

Ten micrograms each of samples A–D reference RNA materials at a concentration of 1 µg/µL were obtained from the FDA sponsored SEQC project (http://www.fda.gov/ScienceResearch/BioinformaticsTools/MicroarrayQualityControlProject/default.htm) [2]. Sample A comprised Stratagene Universal Human Reference RNA (Agilent Technologies Deutschland GmbH, Waldbronn, Germany) mixed with Ambion External RNA Controls Consortium (ERCC) Spike-In Control RNA mix 1 (Life Technologies, Grand Island, New York, USA). Sample B comprised Human Brain Reference RNA obtained from Ambion mixed with ERCC Spike-In Control RNA mix 2. ERCC mixtures 1 and 2 were at a final concentration of 2% in samples A and B, respectively, based on total RNA concentration. Each of the two ERCC RNA spike-in mixes contain the controls spanning a range greater than 10^6^, but in different formulations. Each formulation contains the same four subgroups of controls but for each of the four subgroups there is a fold-difference in concentration between mix 1 and 2; 0.5x, 0.67x, 1.0x and 4.0x-fold respectively (**Dataset S1, S2**). After mixing samples A and B with ERCC mixes 1 and 2, these were combined in 3∶1 and 1∶3 proportional mixtures to create samples C and D, respectively. Thus, samples A-D represent a complex mixture of synthetic (ERCC controls) and endogenous RNA targets in known proportions over a dynamic range greater than 10^6^ and 10^7^, respectively.

### Assay Target Selection

The MicroArray Quality Control (MAQC) consortium (now known as SEQC) previously selected a list of 1,297 genes to evaluate performance of multiple qPCR and microarray platforms [2]. From this list, 150 endogenous targets were selected to develop assays for competitive amplicon library preparation (**Dataset S1**). These 150 assays were chosen, in part, because the gene targets they represent were previously demonstrated to be expressed over a greater than 10^7^ dynamic range across samples A and B. In addition, assays were developed for 28 of 92 ERCC targets (**Dataset S1**). These 28 targets were chosen because they: a) also are present across a large dynamic range (>10^6^) within ERCC formulation mixes 1 and 2, and b) were distributed evenly among the 4 subgroups that exhibit known fold differences in abundance between mix 1 and 2; 0.5x, 0.67x, 1.0x and 4.0x-fold differences [24].

### Reverse Transcription of RNA Reference Materials

#### Reverse transcription 1 (RT1)

For each of samples A–D, two separate 2 µg aliquots of RNA were reverse transcribed in 90 µL volume reactions each using Superscript III reverse transcriptase (Life Technologies) and oligo(dT) priming according to manufacturer’s protocol. After RT, the two 90 µL cDNA products for each sample were combined into a single 180 µL volume.

#### Reverse transcription 2 (RT2)

For sample A, an additional set of two separate 2 µg aliquots of RNA were reverse transcribed in 90 µL volumes and combined. This separate preparation of sample A was used for comparison of inter-library preparation effects.

### Reagent Design

#### Primer design and synthesis

Forward and reverse PCR primers were designed corresponding to 101-bp amplicon regions for each of 150 uniquely transcribed genes in the human genome as well as 28 ERCC targets (**Dataset S1**). Each forward and reverse primer set was designed with a uniform 68°C melting temperature using Primer3 software [25]. Primers with high predicted specificity were selected using GenomeTester 1.3 with human reference genome version 19 to predict off-target amplicons less than 1000 bp in size [26]. Each primer was designed with a universal tail sequence not present in the human genome for multi-template PCR addition of barcode and platform specific sequencing adapters. The forward universal tails were identical to sequence adapters used previously for arrayed primer extension (APEX-2) [27], while the reverse tail sequence was the same as the forward with the exception of the four 3′ bases, enabling directionality during sequencing (**Dataset S1**). Target specific primers with universal tails for the 150 endogenous targets and 28 ERCC targets were synthesized by Integrated DNA Technologies (IDT) and Life Technologies, respectively. Separate primer pools for all endogenous targets or all ERCC targets were created by combining synthesized primers in equimolar ratio and diluting to a final working concentration of 50 nM of each primer in dilute TE buffer (10 mM Tris-Cl, pH 7.4, 0.1 mM EDTA).

#### Competitive internal standard mixture design and synthesis

Each 101-bp competitive IS was designed to retain target specific priming sites identical to its respective native nucleic acid template (**Figure 1A** **and Dataset S1**). Internal to these identical priming sites were six nucleotide changes that enabled differentiation between a competitive IS and its corresponding NT during post-sequencing data analysis (**Figure 1A**). Six nucleotide changes were empirically determined to eliminate post-sequencing data alignment errors of NT versus competitive IS, or vice versa. The 28 competitive IS corresponding to ERCC targets were synthesized by Life Technologies and the 150 competitive IS corresponding to the endogenous targets were synthesized by IDT.

For the 28 competitive IS templates corresponding to ERCC targets, each standard was separately amplified with forward and reverse primers (without universal sequences), column purified (QIAquick PCR purification kit), and visualized and quantified for abundance of full-length templates on an Agilent 2100 Bioanalyzer using DNA Chips with DNA 1000 Kit reagents according to manufacturer’s protocol (Agilent Technologies Deutschland GmbH, Waldbronn, Germany). Quantified standards then were combined at UTMC in a 1∶1 stoichiometric molar ratio, to create a stock concentrated mixture of IS.

The concentration of the competitive IS template for each of the 150 endogenous targets was measured by optical density (OD) at IDT. The IS then were combined into an IS mixture by IDT in a 1∶1 stochiometric molar ratio based on OD measurements. The concentration of each full-length IS in the mixture was empirically determined at UTMC by cross-titrating the competitive IS mixture relative to a constant 100,000 copies (i.e. genome equivalents) of subject ID723 gDNA (**Figure 1B**). We reasoned that in gDNA from a phenotypically healthy individual, the majority of endogenous target loci would be in a 1∶1 proportion to each other, providing a practical and cost-effective reference material to determine the “actual” concentration of full-length amplifiable copies for each endogenous target competitive IS template.

The endogenous target IS mixture and ERCC target IS mixture were each serially diluted to working concentrations and used in all subsequent experiments to quantify copies of each transcript in reverse transcribed samples A-D (**Figure 1**).

### Competitive Amplicon Library Preparation

#### Reaction components

A targeted PCR amplicon sequencing library was prepared from each sample for analysis on an Ion Torrent PGM sequencer using competitive multiplex-PCR (http://ioncommunity.lifetechnologies.com/docs/DOC-7293). For each library, a 10 µL reaction volume was prepared containing: 1 µL of ID 723 gDNA or sample A–D cDNA, 1 µL of competitive IS mixture at varying input concentrations, 1 µL of corresponding primer-mix containing universally tailed target-specific primers, 1 µL of 2 mM dNTPs, 1 µL of 10x Idaho Technology reaction buffer with 30 mM MgCl_2_, 0.1 µL of Promega GoTaq Hot Start Taq polymerase (5 U/µL) and 4.9 µL of RNase free water (**Figure 1**). For quantification of each IS in the 150 endogenous target IS mixture, genomic DNA was spiked into 10 separate competitive multiplex-PCR mixtures containing a) 3-fold serial dilutions, ranging in abundance from 2×10^7^ to 1×10^3^ copies of IS for each gene, and b) primers for the 150 genes. For quantification of each endogenous target in samples A-D cDNA, RT1 and RT2 cDNA from sample A and RT1 cDNA from samples B, C and D were each spiked into 12 separate competitive multiplex-PCRs containing a) three-fold serial dilutions of IS mixture, ranging in abundance from 6×10^7^ - 3.4×10^2^ copies loaded and b) primers for the corresponding genes. A total of 12 µL of each cDNA sample was consumed for endogenous gene analysis, corresponding to 264 ng of RNA for each sample. For analysis of the 28 ERCC targets, RT1 cDNA from samples A–D were each spiked into five PCR reactions containing a) dilutions of IS mixture representing 10^6^, 10^5^, 10^4^, 10^3^ and 300 copies loaded for each of the 28 ERCC targets and b) corresponding primers. A total of 5 µL of each cDNA sample was consumed for ERCC target analysis, corresponding to 110 ng of RNA.

#### Thermal-cycling parameters

As the number of targets in multiplex PCR is increased it is necessary to decrease the concentration of primers used in order to obtain successful first round amplification for all targets [17]. The predominant effect of decreased primer concentration in multiplex PCR is to limit amplicon product formation for the most abundant targets at plateau phase which, in turn, prevents dNTPs from becoming a limiting reagent for less abundant targets [28]. The result is that target templates that vary widely in starting concentration converge toward equimolar concentration at PCR endpoint and this reduces over-sampling or -sequencing of high-abundance targets. Because each target is measured relative to a known number of copies of its respective competitive IS, information regarding the initial proportional representation between native templates is preserved (**Figures 2** **,** **3** **and Animation S1**). However, there is a limit to how much the primer concentration can be diluted and still result in observable amplification product. Dilution of primers, and therefore convergence of template amplicons, was maximized using touchdown PCR and primers with high primer melting temperature [29]. To compensate for low primer concentration and high primer melting temperature, initially high annealing temperatures were incorporated during the earlier cycles of PCR to increase stringency of primer binding and reduce off-target annealing. In subsequent cycles annealing temperature was gradually lowered resulting in increased yield once specific amplicon product was sufficiently formed during earlier higher stringency cycles. Using this framework we developed the following protocol: Each competitive multiplex reaction mixture was cycled in an air thermal cycler (RapidCycler; Idaho Technology, Inc. Idaho Falls, Idaho) for a total of 45 cycles under modified touchdown PCR conditions with low primer concentration: 95°C/3 min (Taq activation); 5 cycles of 94°C/30 sec (denaturation), 72°C/4 min (annealing), and 72°C/15 sec (extension); repeat 5 cycles with annealing temperature decreased 1°C to 71°C; iterate 1°C decrease and 5 cycles until annealing temperature was 64°C. Use of a hot start protocol was absolutely necessary under these conditions to avoid off-target priming that would otherwise result in formation of only primer-dimer products.

**Figure 3:**
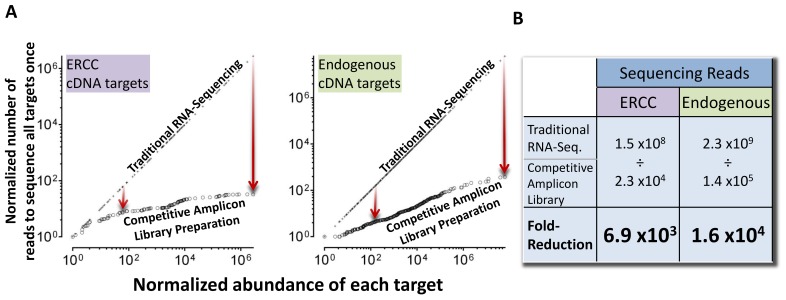
Competitive amplicon library preparation reduced required targeted RNA-sequencing reads up to 10,000-fold. Each sample comprising multiple native targets was mixed in multiple ratios with a competitive internal standard (IS) mixture (depicted in **Figure 1**). The goal is for each combined ratio of native material and competitive IS mixture (**Figure 1A**) to have a subset of targets in “balance” or equivalence (i.e. as close to a 1∶1 relationship of NT:IS as possible). The closer to equivalence, the fewer sequencing reads required across all library preparations to meet criteria outlined in Statistical Methods section for valid measurement of a given target. Preparation of multiple sequencing libraries with competitive IS mixture spiked-in over the range of expression will result in a greater decrease in required sequencing reads than what is obtainable with only one spike-in ratio (as depicted in **Figure 2** and **Animation S1**). **a)** Actual sequencing reads data for 26 ERCC (n = 104) and 100 endogenous (n = 400) cDNA targets across samples A-D. X-axis represents the abundance of each target in a library preparation normalized to the lowest abundance target (set to 10°). Y-axis is in units of normalized sequencing reads (coverage) required to sequence the lowest abundance target at least once. **b)** Tabular summary of panel A where the number of sequencing reads represents the sum of all sequencing reads to observe all targets at least fifteen times. As discussed in the results section, fifteen sequencing reads is sufficient to achieve a type 1 error rate less than 0.05, and a type 2 error rate of less than 0.20. The required number of traditional RNA-sequencing reads is calculated based on an assumed relationship between target copies present prior to library preparation. Fold-reduction in required sequencing reads by competitive amplicon library preparation is the quotient of calculated traditional RNA-sequencing and measured competitive amplicon library preparation sequencing reads.

#### Addition of barcodes and sequencing adapters

A sample from each of the competitive multiplex-PCR reactions was labeled using a unique set of barcode primers. A set of fusion primers containing the barcode sequences and Ion Torrent amplicon sequencing adapters were designed with 1) their 3′-end complementary to the universal APEX-2 sequence tails added during competitive multiplex-PCR, 2) 5′ to that a four nucleotide index/barcode sequence, and 3) 5′ to that, a forward or reverse Ion Torrent amplicon sequencing adapter (**Dataset S1**). Forward and reverse sequencing primers were tailed with different barcodes to dual index each sample and reduce likelihood of false-indexing/barcoding a sequence read [30]. For each barcoding reaction, a 10 µL reaction volume was prepared containing: 1 µL of competitive multiplex-PCR product, 1 µL of 1 uM forward and reverse barcoding primer, 1 µL of 2 mM dNTPs, 1 µL of 10x Idaho Technology reaction buffer with 30 mM MgCl_2_, 0.1 µL of Promega GoTaq Hot Start Taq polymerase (5 u/µL) and 4.9 µL of RNase free water. Each barcoding reaction was cycled in an air thermal cycler (RapidCycler; Idaho Technology, Inc. Idaho Falls, Idaho) under the following conditions: 95°C/3 min (Taq activation); 15 cycles of 94°C/5 sec (denaturation), 58°C/10 sec (annealing), and 72°C/15 sec (extension). Reaction vessels were immediately removed and kept at 4°C during all subsequent steps. The goal during this step was to prevent heterodimerization of barcoded amplification product. Depending on the type of heterodimerization, post-sequencing alignment errors can arise from false base calls during sequencing with resultant decrease in measurement precision and accuracy. These false base calls occur because a single sequencing bead will be populated by two fairly similar heterodimerized templates during emulsion PCR step of Ion Torrent bead preparation (e.g. NT and IS heterodimer for same gene target).

#### Sample multiplexing

Each uniquely barcoded competitive amplicon sequencing library then was individually quantified on an Agilent 2100 Bioanalyzer using DNA Chips with DNA 1000 Kit reagents according to manufacturer’s protocol (Agilent Technologies Deutschland GmbH, Waldbronn, Germany). The sequencing libraries then were mixed in a known stoichiometric ratio to optimize the percentage of sequencing reads that each library would eventually receive; in most cases 1∶1 was used.

#### Product purification and sequencing

It is critical during the purification of barcoded sequencing libraries not to use strong denaturants or chaotropic salts, such as guanidine hydrochloride or thiocyanate. These agents result in downstream template heterodimerization that can result in false base calls and subsequent post-sequencing alignment errors for Ion Torrent sequencing (unpublished studies). For this reason, each mixture of barcoded sequencing libraries was purified using Life Technologies E-Gel SizeSelect 2% Agarose gels, which do not require the use of denaturants or chaotropic salts, and can easily be run in a refrigerated room to prevent heat denaturation during electrophoretic separation. Purified sequencing libraries then were quantified using the Kapa Library Quantification kit for Ion Torrent sequencing platforms (Kapa Biosystems). Based on this quantification, libraries were diluted appropriately and prepared for Ion Torrent PGM Sequencing service according to manufacturer’s recommendations at the University of Toledo Medical Center (UTMC) Department of Pathology, Toledo, OH, and Ohio University (OU) Genomics Facility, Athens, OH.

### Data Processing

#### FASTQ file processing

UTMC and OU Ion Torrent PGM Sequencing services provided raw sequencing data from Ion Torrent Analysis Suite 3.0 in FASTQ format. Sequencing reads greater than 150 bases in length were extracted and each read was parsed into 3 separate FASTQ files: **1)** forward (*query-barcode.fastq*) and **2)** reverse barcode (*query-revbarcode.fastq*) regions (first and last 14 bases of each sequencing read, respectively), as well as **3)** central portion of the amplicon (60 bases) (*query-subject.fastq*) corresponding to the region internal to target specific priming sites where six nucleotide substitutions exist between NT and matching competitive IS.

#### BFAST of sequences against index databases

Each of the three FASTQ files was aligned with FASTA databases (**Dataset S3**) corresponding to whether it was a barcode (*barcode.fa*) or amplicon region (*subject.fa*) using the BLAT-like fast accurate search tool (BFAST, version 0.7.0a), with file output in sequence alignment/map (SAM) format [31]. Input parameters are outlined in supplementary methods (**Methods S1**). BFAST match against the index databases and SAM file output was performed for the trimmed FASTQ files containing 1) forward barcode, 2) reverse barcode and 3) captured amplicon subject sequences.

#### Binning of sequence counts

Each of the three SAM files from 1) forward and 2) reverse barcode, and 3) amplicon region then were merged into a practical extraction and reporting language (PERL) hash table using the sequence read ID as a key for matching (http://www.perl.org/). The PERL scripts for this data processing step are available upon request. Based on barcode and amplicon alignment, each sequencing read was binned into an array corresponding to the IS input concentration for a given sample preparation (**Figure 1**), and whether it was called as a NT or IS by BFAST alignment. If the forward and reverse barcode alignment calls did not match, the sequence read was not binned. The resulting hash table of binned sequencing reads was output in comma delimited format (**Dataset S2,S4**) and processed as outlined in Statistical Methods section.

### Statistical Methods

#### Estimate of native target concentration

For each gene target and technical replicate with input concentration of each IS mixture indexed with the subscript *i*, an estimate of the concentration of the native target (NC_i_) was calculated based on the observed/binned sequence counts of both the native target (NT_i_) and internal standard (IS_i_), as well as the known starting concentration (in units of template copies per library preparation) of the internal standard (SC_i_) (**Dataset S2,S4**):
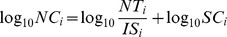



Systematic variation in pipetting of NT and/or competitive IS mixture was identified as a systematic increase or decrease in NC_i_ for all templates for that dilution point technical replicate compared to expected from surrounding dilution point estimates. The median of the systematic difference from expected measured between input concentrations of IS mixture was subtracted from NC_i_ to arrive at the corrected NC**_i_**.

Summarization of the estimates across the technical replicates (NC**_i_**) provided the estimate of the NT concentration (**Figure 1**). Four summarization methods (average, median, least squares, and weighted least squares) were evaluated to estimate the target concentration. The percent variance explained (R^2^) was used as the objective criterion to compare the four methods across a range of the following quality control (QC) parameters corresponding to each transcript: 1) minimum number of sequence counts for an acceptable NT_i_ or IS_i_ measurement; and 2) the inter-replicate coefficient of variation (CV) across NC**_i_**. This search across methods and QC parameter sets was conducted to identify an optimal combination that maximized both the R^2^ measure and the number of transcripts retained. This empirical search determined that an optimal method and QC parameter combination for estimating the summarization quantity was, **1)** the median (NC_median_) of NC**_i_** technical replicate measures that have, **2)** at least 15 sequencing counts for both NT_i_ as well as IS_i_, and **3)** coefficient of variation (CV) across NC**_i_** of less than 1.00 on a base 10 logarithm scale.

#### Methods to assess agreement between estimates of NC_median_

The summarized NC_median_ value for each transcript was compared across different laboratories (e.g. sample A, OU vs. UTMC), days (e.g. sample A, Day 1 vs. Day 2), library preparations (sample A, RT1 vs. RT2), and observed versus expected estimates based on the known mixtures of samples A and B in samples C (75% A and 25% B) and D (25% A and 75% B). Bland-Altman difference plots were used to assess agreement of estimates of NC_median_ and those obtained from known values (for the ERCC samples) and alternative RNA transcript measurement methods (TaqMan qPCR and traditional RNA-sequencing library preparation using Illumina NGS kits [**Dataset S5**]) [32]. Briefly, difference plots display the differences between estimates given by two methods. For this particular application, differences were plotted on the base 10 logarithm scale. Along with the difference plots, scatter plots of the data and corresponding R^2^ values (percent variance explained) from linear models also are displayed. In addition, the area under the receiver operating characteristics (ROC) curve (and corresponding 95% confidence interval) was calculated to assess accuracy in the detection of fold differences (fold changes of 1.10, 1.25, 1.50, 2.00, and 4.00) of ERCC controls known to exist between samples A and B, as well as their derivative mixtures resulting in samples C and D. Results for fold-change ROC curve analysis were binned across differential ratio subpools of pairwise comparisons: 1.1-fold change [1.05–1.174] (controls n = 100, tests n = 96); 1.25-fold change [1.175–1.374] (controls n = 163, tests n = 163); 1.5-fold change [1.375–1.74] (controls n = 229, tests n = 227); 2.0-fold change [1.75–2.49] (controls n = 229, tests n = 223); ≥4.0-fold change [2.5–10.0] (controls n = 286, tests n = 290).

#### Figures

Pairwise comparison plots, Bland-Altman difference plots and ROC curve plots and associated summary statistics were all generated using GraphPad Prism version 6.0. Pairwise comparison linear regression analysis and was performed using a least squares best fit with GraphPad Prism version 6.0. Spearman’s rank correlation analysis was performed using GraphPad Prism version 6.0.

## Results

### Performance Testing of Competitive Amplicon Library Preparation

#### Performance with gDNA

Among the endogenous gene targets, 82% (123 of 150) of designed assays for competitive amplicon library preparation produced one or more valid native target to internal standard (NT:IS) ratio measurements using ID 723 genomic DNA (gDNA) test material (**Figure 1B** and **Table S1**). For those assays with at least triplicate measurements, the ratio of NT:IS sequencing reads decreased in direct proportion to increasing amounts of IS placed into the library preparation (average slope = −1.01x) and at each titration point the inter-gene variation among NT:IS ratios was close to a 1∶1 relationship (**Figure 1B**).

Of the 27 assays with no measurement, 26 had too few sequencing reads consistent with low primer efficiency, and only one (1) assay failed due to unacceptable analytical variation as defined in the Statistics section of Methods.

#### Performance with cDNA

Of the 123 assays that produced valid measurements in gDNA, some did not produce valid measurement in one or more cDNA samples due to low transcript expression and/or low sequencing counts (**Table S3**). Among the four cDNA samples (A–D), a valid measurement was obtained relative to at least one IS concentration for an average of 100 (96–107; samples A–D) of the 123 working endogenous target assays (**Table S1**). For the endogenous targets measurable in cDNA, the ratio of NT:IS sequencing reads decreased in direct proportion to increasing amounts of IS placed into the library preparation (average slope = −0.95x) (**Figure 1B**), as was observed for measurement of gDNA. However, because endogenous cDNA targets were expressed over a >10^7^-fold range, inter-gene variation among NT:IS ratios at each IS cross-titration point was much greater than that observed with gDNA (**Figure 1B**).

Among the ERCC targets, 26 of 28 designed assays produced one or more measurements in samples A–D (**Table S1**). For one assay (ERCC 46) sequencing reads were too few for both NT and IS across samples A-D indicating low PCR efficiency, and only one assay (ERCC 96) failed due to failure to titrate NT:IS. For the 26 successful assay measurements, the average slope of cross-titrating NT with competitive IS was −1.02x (**Figure 1B**).

### Competitive Amplicon Library Preparation Reduced Oversampling in RNA-sequencing

Dynamic range of a method can be defined as the fold difference from highest to lowest measureable value. The observed dynamic range measured as transcript copies per competitive amplicon library preparation was 2.7×10^6^-fold for the 26 ERCC targets and 6.2×10^7^-fold for the approximately 100 endogenous cDNA targets assessed across samples A–D. The number of sequencing reads required to sequence all targets at least fifteen times was 2.3×10^4^ and 1.4×10^5^, respectively, for the same 26 ERCC and 100 endogenous cDNA targets (**Figure 3**). We chose fifteen (15) sequencing reads as a benchmark for quantification because it represents sufficient sampling of a given target to enable the detection of a 2-fold change in abundance between targets with a type 1 error rate of less than 0.05, and a type 2 error rate less than 0.20. It is important to note that it is assumed that the precision of quantification in RNA-sequencing, targeted or not, is directly proportional to the number of sequencing reads a given transcript or amplicon has, and that the sampling of low abundance targets fits a Poisson distribution. This assumption may not be true under some conditions as discussed below [24].

For “traditional” RNA-sequencing library preparation methods, the starting proportionality among native targets must be maintained during library preparation in order to achieve reproducible quantification. Assuming that this proportionality is maintained, the total number of sequencing reads required for traditional RNA-sequencing can be calculated as the sum of sequencing reads of all targets that is required to sequence the least abundant target at least fifteen times. In a simplified example of this calculation, if two transcript targets of interest are expressed over a 1 million fold dynamic range, 15 million plus 15 sequencing reads (15,000,015 sequencing reads) would be required in each traditional RNA-sequencing library preparation technical replicate to have sufficient statistical significance to detect a two-fold change in transcript abundance between targets. This summated number of required sequencing reads is much larger when more than two transcript species are present in a given sample.

**Figure 4:**
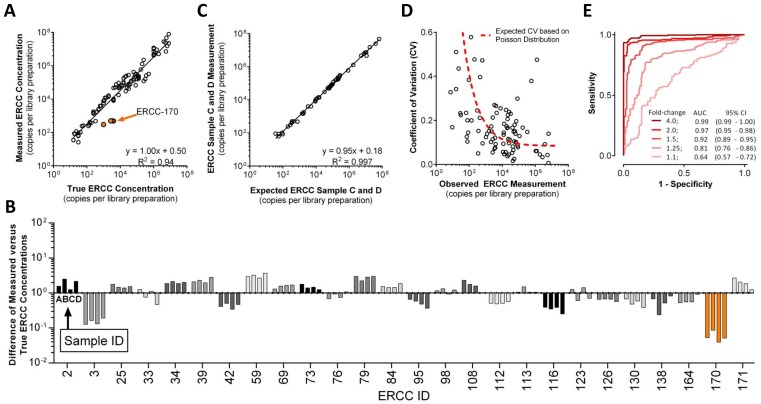
Performance of competitive amplicon library preparation with ERCC Reference Materials. **a)** Measured signal abundance of ERCC targets in samples A, B, C and D. X-axis units are derived from Ambion product literature for the known concentration of ERCC spike-in controls (n = 104). **b)** Difference plots of data in panel A ordered numerically by ERCC ID. Each ERCC target depicted was measured at least once in all four samples A–D. For purposes of clarity, ERCC-170 is highlighted orange in panels A and B (n = 104). **c)** Samples C and D represent a 3∶1 and 1∶3 mixture, respectively, of samples A and B. These ratios were used to calculate expected measurements for samples C and D (X-axis). Actual measurements of samples C and D are plotted on the Y-axis (n = 52). **d)** Coefficient of variation (CV) in measurements of ERCC targets in samples A-D, for those assays with at least two IS dilution points. Red line depicts expected CV based on a Poisson sampling (n = 95). **e)** ROC curves to detect fold change with corresponding area under the curve (AUC) with 95% confidence intervals. ROC curves are derived from the comparison of differential ratio subpools of ERCC targets in samples: A vs. B, A vs. C, A vs. D, B vs. C, B vs. D and C vs. D. Results for 1.1-fold change represent a range of differential ratio subpools [1.05–1.174] (controls n = 100, tests n = 96); 1.25-fold change [1.175–1.374] (controls n = 163, tests n = 163); 1.5-fold change [1.375–1.74] (controls n = 229, tests n = 227); 2.0-fold change [1.75–2.49] (controls n = 229, tests n = 223); ≥4.0-fold change [2.5–10.0] (controls n = 286, tests n = 290).

As written above, clarification is required, as it is somewhat difficult to compare sequencing reads obtained from traditional RNA-sequencing versus PCR-based targeted RNA-sequencing approaches. A key distinguishing feature is that the former has a component of transcript length and percentage of transcript sequenced by at least a certain number of reads (i.e. sequencing coverage), while the latter targeted PCR approach simply requires counting the number of times a specific targeted region is sequenced (i.e. sequencing reads). As an example of the differences, in traditional RNA-sequencing a single RNA molecule of 5 kilobases in length may be fragmented into 50 separate ∼100 base length RNA molecules that when processed through ligation steps and sampled with sequencing may not exhibit a similar sampling distribution as smaller RNA molecules. In contrast, for targeted RNA-sequencing with PCR-based library preparation, transcript length should not impact sampling distribution phenomena because fragmentation is not typically involved. This difference may account for some of the deviations from Poisson distribution sampling laws previously observed in traditional RNA-sequencing methods [24] versus observations of a good fit to Poisson sampling in our targeted approach (**Figure 4C**). Thus, the authors recognize there are a number of limitations in a direct comparison between our method, traditional RNA-sequencing, and some targeted RNA-sequencing approaches that still require native template fragmentation. That being stated, a careful comparison of sequencing counts between methods is still possible.

Based on the assumptions outlined, for traditional RNA-sequencing library preparation methods the calculated number of reads required to sequence all targets at least fifteen times was 1.5×10^8^ and 2.3×10^9^, for the 26 ERCC and 100 endogenous cDNA targets respectively (**Figure 3B**). This approximation is based on the sum of measured transcript copies using competitive amplicon library preparation method with the least abundant target sequenced at least 15 times. Notably, these calculations closely approximate the actual sum of sequencing reads (∼5.0×10^9^) required to sequence the same 100 endogenous targets at least fifteen times each using traditional RNA sequencing methods in preliminary SEQC project study data (**Dataset S5**). For our comparative uses of the SEQC preliminary data, it was not relevant if the 15 sequencing reads were in the same location of a specific transcript or 15 sequencing reads distributed across the length of a transcript; they only needed to be at least 15 read counts with sequence unique to that transcript. Thus, compared to traditional RNA-sequencing, targeted quantitative RNA-sequencing with competitive amplicon library preparation decreased required sequencing reads from 1.5×10^8^ and 2.3×10^9^ to 2.3×10^4^ and 1.4×10^5^, respectively, for the 26 ERCC and 100 endogenous cDNA targets; an average decrease in required reads of 1.15×10^4^-fold (6.9×10^3^ to 1.6×10^4^) for transcripts expressed over a >10^6^ fold dynamic range (**Figure 3B**). This observed reduced sequencing read requirement compared to traditional or other targeted quantitative RNA-sequencing methods can be primarily attributed to the intentional convergence (i.e. normalization) of abundance among amplicons during plateau phase of competitive multiplex PCR-driven library preparation.

### Performance of Competitive Amplicon Library Preparation with ERCC Reference Materials

There was high correlation (R^2^ = 0.94; **Figure 4A**) between expected ERCC target copy concentration, based on Ambion reported input ERCC target concentrations (**Dataset S2**), and measured copy numbers per library preparation. The observed slope and intercept (1.00x +0.50)_log10_ indicated no signal compression and good agreement between measured and expected cDNA molecules based on an assumed 100% efficiency in conversion of ERCC RNA to cDNA during reverse transcription. The median *intra*-assay ERCC measurement coefficient of variation (CV) was 20% across each sample’s technical replicates and 19% across samples A–D (**Table S2**). As noted in the methods, samples C and D represent a known cross-mixture of total RNA from samples A and B. Thus measurements made in A and B were used to calculate expected measurements for samples C and D (e.g. 0.75*A +0.25*B to predict C; and 0.25*A +0.75*B to predict D). Based on these calculations, there was excellent correlation, and thus reproducibility, between expected and measured for Samples C and D (R^2^ = 0.997) (**Figure 4C**).

The *intra-sample intra-assay* CV for technical replicates progressively increased as the copies loaded per library preparation decreased below ∼10^3^ (**Figure 4D**). This measured increase in CV corresponds well to expected increase in CV based on a Poisson distribution and the stochastic process of sampling low abundances of cDNA molecules during library preparation. Thus, the majority of assay variance at low copy number is due to sampling variance dictated by natural law, and not platform-specific technical variance.

To evaluate accuracy to detect a given fold-change in abundance between samples, the defined ratios between sub-pools of ERCC controls in mixes 1 and 2, which were spiked into samples A and B respectively at known concentrations, were used as truth in receiver operating characteristic (ROC) curve analysis (SEQC main study). Moreover, since samples C and D represent known cross-mixtures of A and B, the factorial comparison of possible pair-wise tests to detect fold-change between control (n = 1007) and test (n = 999) measurements for each sub-pool allowed for fine resolution discrimination analysis of expected and observed fold-change (e.g. sample A vs. B, A vs. C, A vs. D, B vs. C, B vs. D and C vs. D). The ROC analysis area under the curve (AUC) provided a measure of relative accuracy to detect a fold-difference between control and test groups of measurements. Using this approach competitive amplicon library preparation had a 97% accuracy (AUC) to detect a two-fold change in ERCC target abundance between samples, with commensurate increase or decrease in accuracy with higher or lower expected fold-change, respectively (**Figure 4E**).

### Performance of Competitive Amplicon Library Preparation for Measurement Endogenous cDNA Targets

**Figure 5:**
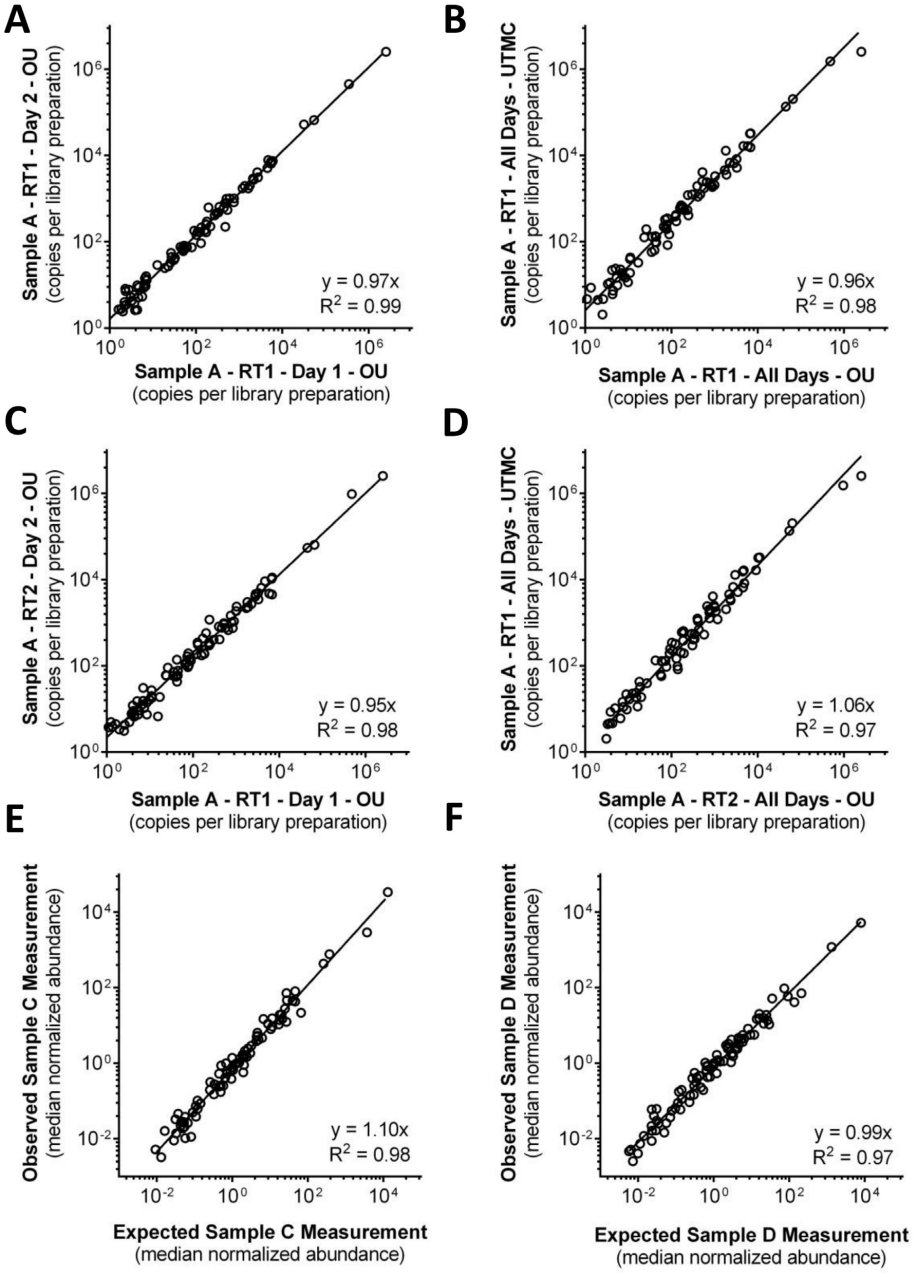
Performance of competitive amplicon library preparation with endogenous cDNA targets. **a–d)** Absolute signal abundance of cDNA targets in sample A in units of copies per library preparation measured on separate days, at different sites (OU = Ohio University; UTMC = University of Toledo Medical Center), and between different reverse transcription preparations (RT1 and RT2). **a)** Inter-day effect (n = 88). **b**) Inter-day and Inter-site effect (n = 81). **c)** Inter-day and Inter-library effect (n = 92). **d)** Inter-day, Inter-site and Inter-library effect (n = 80). **e–f)** Samples C and D represent a 3∶1 and 1∶3 mixture, respectively, of total RNA from samples A and B. These ratios were used to calculate expected measurements for samples C and D (X-axis) from measurements of A and B. Plotted on the Y-axis are actual measurements of samples C (n = 86) and D (n = 90).

The reproducibility of endogenous transcript abundance measurements using competitive amplicon library preparation was assessed using correlation coefficients from pairwise comparisons of endogenous transcript abundance measurements on separate days with the same library and sequencing site (**Figure 5** **, panel A**; R^2^ = 0.99), separate days and sites with the same library (**panel B**; R^2^ = 0.98), separate day and library preparation with same sequencing site (**panel C**; R^2^ = 0.98) and separate day, site and library preparation (**panel D**; R^2^ = 0.97). Analysis of library preparation effects includes separate reverse transcriptions (RT1 and RT2).

Because samples C and D represent a known cross-mixture of A and B, measurements made in A and B, can be used to calculate expected measurements for C and D (**Figure 4E,F**). Observed correlation (R^2^) between expected and measured for samples C and D was 0.98 and 0.97 respectively.

### Cross-platform Comparison with Competitive Amplicon Library Preparation Measurements

For the vast majority (∼90%) of endogenous target measurements the difference in results measured in this study compared to results previously reported for TaqMan qPCR and for traditional RNA-sequencing library preparation using Illumina next-generation sequencing (NGS) kits (http://www.fda.gov/ScienceResearch/BioinformaticsTools/MicroarrayQualityControlProject/default.htm) was systematic across all samples A–D (**Figures S1, S2**) [2]. For a smaller fraction (∼10%), bias was highly different in sample A versus B for both TaqMan qPCR and traditional RNA-Sequencing with Illumina kit (e.g. gene IDs - BAG1, ELAVL1, SOX15 and others). Moreover, in this smaller subset of assay targets, an intermediate level of systematic bias was observed in samples C and D compared to A and B. This intermediate level of systematic bias likely is due to the fact that samples C and D are cross-titrations of samples A and B. This trend was not observed in any of the ERCC reference RNA controls assessed (**Figure 4B**). This finding indicates that a small number of endogenous target assays, for each platform, measured a unique signal specific to sample A or B; most likely cross-platform differences in targeting transcript isoforms, as was noted for ELAVL1 in MAQC I study [2].

**Figure 6:**
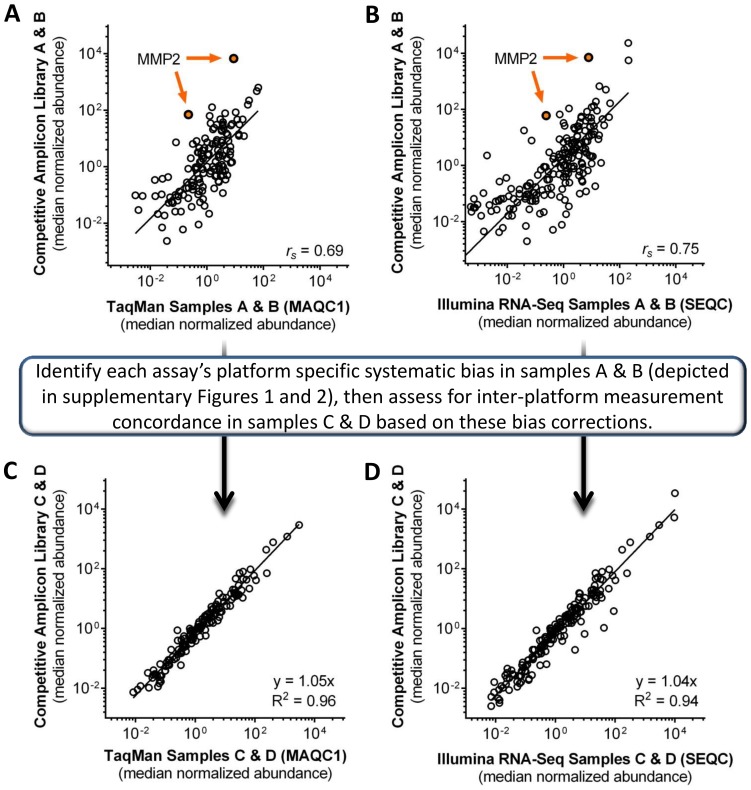
Cross-platform comparison of competitive amplicon library preparation with TaqMan qPCR and Illumina RNA-Sequencing. **a**) Comparison of TaqMan qPCR with competitive amplicon library preparation (n = 146) for samples A and B without correction for systematic biases. Data is normalized to a median relative abundance. **b**) Comparison of Illumina RNA-Sequencing with competitive amplicon library preparation (n = 170) for samples A and B without correction for systematic biases. Data is normalized to a median relative abundance. For a) and b), Spearman’s rank correlation coefficient is noted (r_s_). The average of differences for measurements of samples A and B between competitive amplicon library preparation and TaqMan qPCR (**Figure S1**) or Illumina RNA-sequencing (**Figure S2**) was determined for each endogenous target; and to illustrate the systematic bias away from the regression line, data points for MMP2 have been highlighted in orange. This difference was subtracted from TaqMan qPCR or Illumina RNA-sequencing measurements for samples C and D and plotted (X-axis). Competitive amplicon library preparation measurements of C and D are plotted on the Y-axis. **c)** Comparison of TaqMan qPCR with competitive amplicon library preparation (n = 146) for samples C and D with correction for platform and assay specific bias. **d)** Comparison of Illumina RNA-Sequencing with competitive amplicon library preparation (n = 170) for samples C and D with correction for platform and assay specific bias.

Spearman’s rank correlation analysis for samples A and B is significant between competitive amplicon library preparation method and TaqMan qPCR (r_s_ = 0.69; p<0.005) as well as traditional RNA-Sequencing with Illumina kit (r_s_ = 0.75; p<0.005) (Figure 6A,B). For each assay, in each platform, a systematic difference was observed (e.g. MMP2; Figure 6A,B). These observed systematic differences for A and B (**Figures S1, S2**) were averaged and subsequently subtracted away from the raw reported measurements for TaqMan qPCR or traditional RNA-sequencing measurements for samples C and D. These TaqMan- and Illumina-corrected measurements of C and D were plotted against measurements of C and D obtained with competitive amplicon library preparation (**Figure 6C,D**). This approach and comparison was taken for several reasons. Principally, there are large systematic measurement differences between the method reported here and TaqMan qPCR or traditional RNA-sequencing in samples A and B (**Figures S1, S2**). This systematic difference was largely recapitulated in samples C and D. We reasoned that these differences were due to how each platform interpreted the assay signal for each target. As an example, the measured signal for a given target in traditional RNA-sequencing is filtered through a set of biases such as GC content, transcript length, transcript fragmentation efficiency during library preparation, ligation of sequencing adapters and so forth. These systematic effects can be large at times [33]. In this inter-platform comparison we sought to demonstrate that once these large differences were corrected for, both our method as well as TaqMan qPCR and traditional RNA-sequencing will be largely concordant for absolute transcript abundance measurements in Samples C and D. Based on these pairwise comparisons, we observed a high degree of cross-platform agreement between competitive amplicon library preparation targeted RNA-sequencing with TaqMan qPCR (R^2^ = 0.96), as well as Illumina RNA-Sequencing (R^2^ = 0.94) for absolute expression measurements. It is highly likely that additional correction for transcript isoform differences could result in a higher degree of concordance. However, this transformation was not attempted in order to avoid over-fitting of the data.

The caveat for interpreting data from this inter-platform comparison is to understand that there is not an accepted gold-standard for nucleic acid measurement in complex mixtures of endogenous targets as compared to the more straightforward analysis of ERCC cDNA performed above. This is further compounded by platform differences in targeting of naturally occurring endogenous transcript isoforms which are more likely the rule than the exception (unpublished SEQC studies). Because of this, a comparison of fold-change detected between platforms using endogenous materials is frought with difficulties in parsing out the effects of the platform, and the resulting intrinsic differences in how each transcript signal is interpreted in each platform. Thus, the most important message in this inter-platform comparison is that there can be a large degree of agreement between platforms’ absolute expression measurements, when systematic bias is accounted for.

## Discussion

Based on data presented here, targeted RNA-sequencing of competitive amplicon library preparations provides a quality-controlled method suitable for transcript-based biomarker development and implementation, including collection of data suitable for the clinical setting as well as submission to regulatory agencies. Numerous regulatory agencies, consensus groups and investigators have recommended that nucleic acid based *in-vitro* diagnostic devices, if applicable, should include quality controls for non-systematic analytical variation and false negative reporting [15], [16]. Of note, competitive IS have been used for more than two decades to provide necessary quality control for PCR-based *in vitro* nucleic acid diagnostic devices [13], [22]. Competitive IS use in PCR enables accurate and precise quantification at amplification plateau phase and use of the same competitive IS mixture ensures inter-laboratory concordance of results [17]–[19]. In this study, we apply competitive IS mixtures to targeted amplicon sequencing of RNA derived templates (**Figure 1**). Using ERCC reference materials of known abundances and proportions (**Dataset S2**; **Figure 4**) [24], we demonstrate excellent reproducibility (R^2^ = 0.997) with 97% accuracy to detect 2-fold change (**Figure 4**). In addition, results support utility of this method in reducing required sequencing reads by driving amplicon abundances (through normalization) toward equimolar proportions at plateau phase of library preparation, resulting in a 10,000-fold reduction in required sequencing reads (**Figures 2** **–** **3** **and Animation S1**), without compressing the linear dynamic space for signal measurement by utilizing competitive internal standard reference templates (**Figures 4** **–** **6**).

Consistent with previous reports [2], [24], systematic differences from expected measurements were observed (**Figures 4AB, S1** and **S2**). Possible reasons for observed systematic differences in measurements of synthetic ERCC targets include, but are not limited to: 1) concentrations different from those reported in product documentation due to the multi-step process of creating the ERCC RNA targets in mixes 1 and 2 to prepare samples A–D, 2) systematic differences in reverse transcription efficiency between ERCC targets, and 3) variation introduced during the multi-step process of preparing the competitive IS mixture for ERCC targets used in this study. Observed inter-platform variation in measurement of endogenous gene targets (i.e. competitive amplicon library preparation targeted RNA-sequencing vs. TaqMan vs. traditional RNA-sequencing on Illumina NGS) was largely systematic for each target (**Figures S1, S2**). One source of systematic variation between methods may be ligation bias exhibited during more traditional RNA-sequencing methods, which can be as large as 1000-fold [33]. One possible source of non-systematic inter-platform variation in measurement of a select number of endogenous assay targets is that different transcript isoforms were assessed by the different platforms. This likely is the mechanism for targets where the direction of variation is different for sample A than it is for sample B as is evident for BAG1, ELAVL1, SOX15 and others (**Figures S1, S2**) [2] (also observed in preliminary SEQC studies). Another reason for systematic differences may relate to inter-platform variation in RT methods used. Notably, because the majority of differences are systematic, they do not cause inter-platform variation in absolute expression measurement for those assays that are targeting the same transcript isoforms (**Figures S1, S2 and 6**). This study compared accuracy to detect fold-change in ERCC targets, but not for endogenous cDNA targets. The reasoning here is that for endogenous RNA targets, no gold-standard for truth in measuring fold-change has been established (SEQC preliminary results main study). However, a comparison between the fold-change observed with a method, and that expected based on the known composition and abundance of each ERCC target, is a very good measure of analytical performance (**Figure 4**).

For routine molecular diagnostic tests that measure nucleic acids, it is important to have well-characterized set of quality-controlled assays that focus on a specific set of clinically relevant questions. The method presented here reduces the data complexity and costs in a number ways, making it easier for the implementation of focused quantitative sequencing panels in the clinical setting. The chief advantage of this method relative to a typical qPCR clinical diagnostic is reduced cost, qualitative sequencing information and simultaneous measurement of a large number of targets per technical replicate with minimal sample usage. The authors do recognize the need, at times, for whole-transcriptome sequencing for discovery of the occasional clinically relevant transcript alteration that is not routinely assayed. However, in this study we chose to address a separate but equally important need for a method, which enables reproducible quantitative sequencing panels that cost-effectively assess routine clinical entities.

The cost and time for synthesizing competitive IS mixtures is coming down rapidly due to array based synthesis methods [34], as is sequencing cost per read. These trends will enable routine, cost-effective development and implementation of large competitive amplicon sequencing panels. Further, the use of competitive IS mixtures potentially may be adapted to even larger scale targeted RNA-sequencing approaches; such as those performed using array- or solution-based bait libraries [3], [6], or even molecular inversion capture probes [7].

## Conclusions

We describe competitive multiplex PCR amplicon library preparation for targeted quantitative RNA-sequencing, which 1) provides quantitative transcript abundance data sets for selected gene targets that are concordant across days, library preparations and laboratories, and 2) reduces sequencing reads required for transcript abundance quantification by more than 10,000-fold.

### Data Sccess

All data generated in this study are available online in **Datasets S1–5**.

## Supporting Information

Figure S1 **Difference plots between TaqMan qPCR and competitive amplicon library preparation based measurements.**
(PDF)

Figure S2 **Difference plots between Illumina RNA-sequencing and competitive amplicon library preparation based measurements.**
(PDF)

Table S1 **Competitive amplicon library preparation qualitative assay performance.**
(PDF)

Table S2 **Coefficient of variation (CV) of ERCC measurements.**
(PDF)

Table S3 **“True negative” calls: competitive amplicon library preparation versus TaqMan qPCR and Illumina RNA-sequencing.**
(PDF)

Dataset S1 **Reagent design.**
(XLS)

Dataset S2 **ERCC targets sequencing counts.**
(XLS)

Dataset S3 **FASTA database index input.**
(XLS)

Dataset S4 **Endogenous targets sequencing counts.**
(XLS)

Dataset S5 **Cross-platform comparison data.**
(XLS)

Methods S1 **BFAST input parameters.**
(DOCX)

Animation S1 **Animated illustration of how competitive internal standard templates enable reproducible quantification, and controls for normalization of native template amplicons during PCR-based sequencing library preparation, thus leading to reduced required sequencing counts for accurate quantification.**
(PPTX)
