# Correction: Targeted RNA-Sequencing with Competitive Multiplex-PCR Amplicon Libraries

**DOI:** 10.1371/annotation/f77a351e-f57b-4102-b80c-6c4507beaba6

**Published:** 2013-12-03

**Authors:** Thomas M. Blomquist, Erin L. Crawford, Jennie L. Lovett, Jiyoun Yeo, Lauren M. Stanoszek, Albert Levin, Jia Li, Mei Lu, Leming Shi, Kenneth Muldrew, James C. Willey

The number of one of the grants from the National Cancer Institute is incorrect. The Funding Statement should read: "Significant portions of this study were paid for with funding provided by National Institutes of Health, National Cancer Institute (RC2-CA148572 and IMAT R21-CA138397) and National Heart Lung and Blood Institute (RO1-HL108016); and the University of Toledo Medical Center George Isaac Research Fund. The funders had no role in study design, data collection and analysis, decision to publish, or preparation of the manuscript." 

