# Supplementary material for: Targeted RNA-Sequencing with Competitive Multiplex-PCR Amplicon Libraries
Source: PLoS One. 2013 Nov 13;8(11):e79120. doi: 10.1371/journal.pone.0079120 (PMC3827295; doi:10.1371/journal.pone.0079120)

Supplementary Figure 2. Difference plots between Illumina RNA-Sequencing and and competitive amplicon library preparation based measurements.

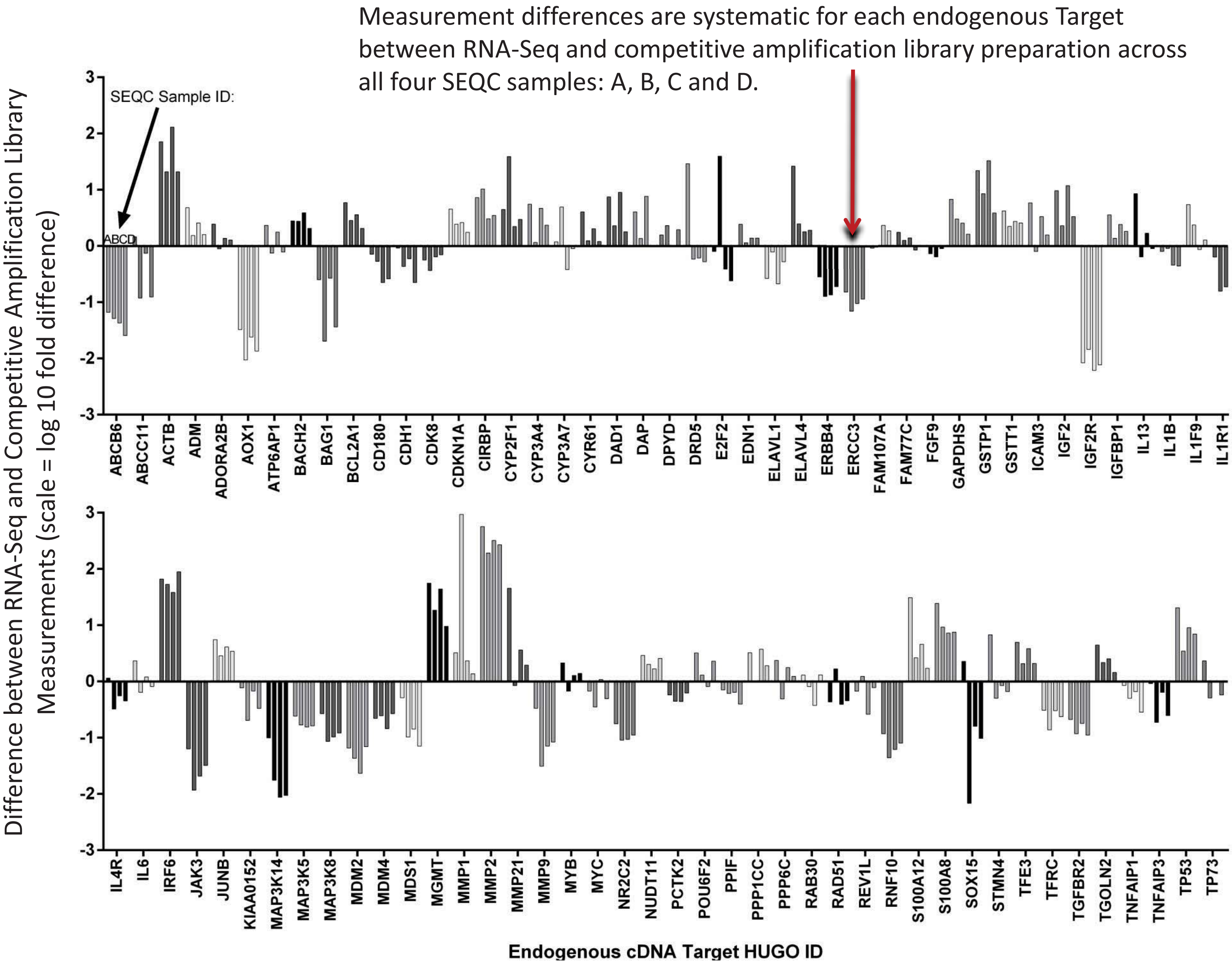

Supplement: Figure S2 — Difference plots between Illumina RNA-sequencing and competitive amplicon library preparation based measurements. (PDF) [file pone.0079120.s002.pdf]
