# Supplementary material for: Targeted RNA-Sequencing with Competitive Multiplex-PCR Amplicon Libraries
Source: PLoS One. 2013 Nov 13;8(11):e79120. doi: 10.1371/journal.pone.0079120 (PMC3827295; doi:10.1371/journal.pone.0079120)
Supplement: Table S1 — Competitive amplicon library preparation qualitative assay performance. (PDF) [file pone.0079120.s003.pdf]

Supplementary Table 1. Assay Performance

ERCC Target Measurements	A	B	C	D
	Sample ID			
**Valid**	26	26	26	26
**True negative**	0	0	0	0
**Failed**	1	1	1	1

Endogenous Target Measurements	A	B	C	D	gDNA
**Valid**	99	99	96	107	123
**True Negative**	4	12	6	4	0
**Failed**	20	12	21	12	0

Assay measurement performance as assessed in samples A-D for ERCC as well as endogenous cDNA targets. Endogenous targets were also assessed against gDNA control (depicted in Figure 1B).

True negative measurements occur when sufficient number of competitive internal standard was sequenced (sequenced at least 15 times), but insufficient native template was observed across all spike-in concentrations of internal standard. An upper limit of expression for these assays can still be calculated as [1/(IS sequencing counts)] x concentration IS loaded into the library preparation with the lowest IS concentration present. These measurements represent true negative measurements and the lower limit of accurate quantification can be determined from these data. In this way, competitive IS mixtures can control for false negative reporting.

Failed assays are measurements where “sequencing depth was too low” for both the NT and IS. These represent true assay failures (neither native or internal standard was sequenced at least 15 times). Increasing sequencing coverage will recover these assays and provide valid measurements.
