# Supplementary material for: Targeted RNA-Sequencing with Competitive Multiplex-PCR Amplicon Libraries
Source: PLoS One. 2013 Nov 13;8(11):e79120. doi: 10.1371/journal.pone.0079120 (PMC3827295; doi:10.1371/journal.pone.0079120)
Supplement: Table S2 — Coefficient of variation (CV) of ERCC measurements. (PDF) [file pone.0079120.s004.pdf]

Supplementary Table 2. Coefficient of variation (CV) of ERCC measurements.

**Intra-assay; Intra-sample CV**	0.20
**Intra-assay; Inter-sample CV**	0.19

CV of differences is calculated from data presented in Figure 4. CV = (Standard Deviation of measurements)/(mean of measurements)

Intra-assay Intra-sample CV is calculated from the median of intra-assay CV within each sample A-D.

Intra-assay Inter-sample CV is calculated from the median of intra-assay CV across samples A-D.
