# Supplementary material for: Targeted RNA-Sequencing with Competitive Multiplex-PCR Amplicon Libraries
Source: PLoS One. 2013 Nov 13;8(11):e79120. doi: 10.1371/journal.pone.0079120 (PMC3827295; doi:10.1371/journal.pone.0079120)
Supplement: Table S3 — “True negative” calls: competitive amplicon library preparation versus TaqMan qPCR and Illumina RNA-sequencing. (PDF) [file pone.0079120.s005.pdf]

Supplementary Table 3. "True negative" calls: competitive amplicon library preparation versus Taqman and RNA-sequencing.

HUGO and Sample ID	CALP	Taqman	RNA-Seq
RPL3L Sample-D	< -2.4879	-3.24	-2.90
ANXA13 Sample-C	< -2.127	-3.20	-2.25
MMP7 Sample-C	< -1.7003	-3.27	-2.23
KRT24 Sample-D	< -1.6589	-3.00	-2.31
KIAA0101 Sample-B	< -1.3856	-2.24	-1.64
KRT24 Sample-B	< -1.2609	-2.83	-1.88
STMN2 Sample-A	< -1.0849	-1.47	-1.92
IL18R1 Sample-B	< -0.938	-2.28	-0.81
DPP4 Sample-B	< -0.733	-1.64	-0.61
IL18R1 Sample-D	< -0.7132	-2.37	-1.10
KIT Sample-C	< -0.0946	-0.80	-0.18
KIT Sample-A	< 0.1187	-1.21	-0.53
IL1F6 Sample-B	< -1.9726	ND	ND
POU1F1 Sample-C	< -1.9169	ND	-3.95
POU1F1 Sample-B	< -1.8552	ND	-2.62
RPL3L Sample-B	< -1.8183	ND	-3.15
KRT24 Sample-C	< -1.6485	ND	-2.72
POU1F1 Sample-A	< -1.5431	ND	-4.18
CYP2C9 Sample-D	< -1.1907	ND	-3.41
ABCB11 Sample-B	< -1.1123	ND	-2.38
SERPINB7 Sample-B	< -1.0772	ND	-3.15
DLG7 Sample-B	< -0.8081	ND	-2.31
FOXA1 Sample-B	< -0.4969	ND	-2.42
CYP2C9 Sample-C	< 0.37	ND	-3.20
CYP2C9 Sample-B	< 0.6952	ND	-2.92
CYP2C9 Sample-A	< 0.7906	ND	-3.16

26 competitive amplicon library measurements had sufficient data to report back a less than measurement. Of the 26 measurements, TaqMan reported not detected (ND) for 14, and RNA-Seq reported ND for 1. Because competitive amplicon library preparations could detect IS, but not accurately quantify NT present, these represent False Negative detections for TaqMan and RNA-Seq. Less than measurements were calculated as [1/(IS sequencing counts)] x concentration IS loaded into the library preparation.
