# Supplementary material for: Targeted RNA-Sequencing with Competitive Multiplex-PCR Amplicon Libraries
Source: PLoS One. 2013 Nov 13;8(11):e79120. doi: 10.1371/journal.pone.0079120 (PMC3827295; doi:10.1371/journal.pone.0079120)
Supplement: Methods S1 — BFAST input parameters. (DOCX) [file pone.0079120.s011.docx]

**BFAST input parameters:**

BFAST database indices were seeded for barcode sequence alignments in a POSIX-compliant environment (see barcode.fa in supplementary materials):

*bfast fasta2brg -f barcode.fa && bfast index -f barcode.fa -m 1111111111 -w 10 -i 1 && bfast index -f barcode.fa -m 1111111101 -w 9 -i 2 && bfast index -f barcode.fa -m 1111111011 -w 9 -i 3 && bfast index -f barcode.fa -m 1111110101 -w 8 -i 4 && bfast index -f barcode.fa -m 1111101011 -w 8 -i 5 && bfast index -f barcode.fa -m 1111010101 -w 7 -i 6 && bfast index -f barcode.fa -m 111010101 -w 6 -i 7 && bfast index -f barcode.fa -m 1110101 -w 5 -i 8 && bfast index -f barcode.fa -m 11101101 -w 6 -i 9 && bfast index -f barcode.fa -m 11111111 -w 8 -i 10*

BFAST database indices were seeded for amplicon subject sequence alignments in a POSIX-compliant environment (see subject.fa in supplementary materials):

*bfast fasta2brg -f subject.fa && bfast index -f subject.fa -m 11111111 -w 8 -i 1 && bfast index -f subject.fa -m 111101111 -w 8 -i 2 && bfast index -f subject.fa -m 111011111 -w 8 -i 3 && bfast index -f subject.fa -m 111110111 -w 8 -i 4 && bfast index -f subject.fa -m 110111111 -w 8 -i 5 && bfast index -f subject.fa -m 111111011 -w 8 -i 6 && bfast index -f subject.fa -m 1011111101 -w 8 -i 7 && bfast index -f subject.fa -m 1100111111 -w 8 -i 8 && bfast index -f subject.fa -m 1111001111 -w 8 -i 9 && bfast index -f subject.fa -m 1111110011 -w 8 -i 10*

BFAST match against the index databases and SAM file output was performed for the trimmed FASTQ files containing 1) forward barcode, 2) reverse barcode and 3) captured amplicon subject sequences in a POSIX-compliant environment as follows:

*bfast match -f barcode.fa -r query-barcode.fastq -n 8 -l > bfast-barcode-matches.bmf && bfast localalign -f barcode.fa -m bfast-barcode-matches.bmf -n 8 > bfast-barcode-aligned.baf && bfast postprocess -f barcode.fa -i bfast-barcode-aligned.baf -n 8 -a 3 > bfast-barcode-report.sam*

-and-

*bfast match -f barcode.fa -r query-revbarcode.fastq -n 8 -l > bfast-revbarcode-matches.bmf && bfast localalign -f barcode.fa -m bfast-revbarcode-matches.bmf -n 8 > bfast-revbarcode-aligned.baf && bfast postprocess -f barcode.fa -i bfast-revbarcode-aligned.baf -n 8 -a 3 > bfast-revbarcode-report.sam*

-and-

*bfast match -f subject.fa -r query-subject.fastq -n 8 -l > bfast-subject-matches.bmf && bfast localalign -f subject.fa -m bfast-subject-matches.bmf -n 8 > bfast-subject-aligned.baf && bfast postprocess -f subject.fa -i bfast-subject-aligned.baf -n 8 -a 3 > bfast-subject-report.sam*
