# Supplementary material for: Targeted RNA-Sequencing with Competitive Multiplex-PCR Amplicon Libraries
Source: PLoS One. 2013 Nov 13;8(11):e79120. doi: 10.1371/journal.pone.0079120 (PMC3827295; doi:10.1371/journal.pone.0079120)
Supplement: Animation S1 — Animated illustration of how competitive internal standard templates enable reproducible quantification, and controls for normalization of native template amplicons during PCR-based sequencing library preparation, thus leading to reduced required sequencing counts for accurate quantification. (PPTX) [file pone.0079120.s012.pptx]

## Slide 1
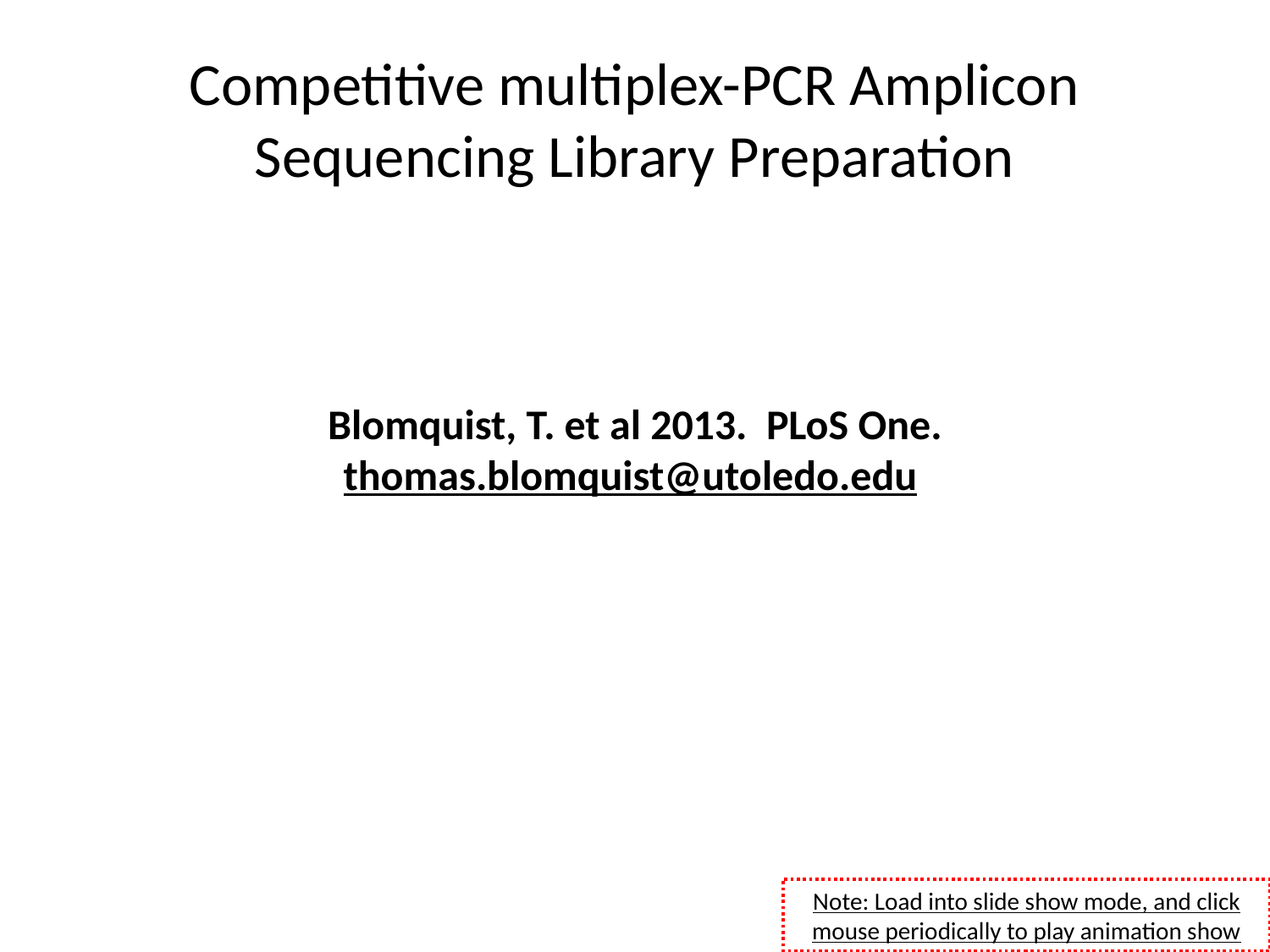

Competitive multiplex-PCR Amplicon Sequencing Library Preparation
Blomquist, T. et al 2013. PLoS One.
thomas.blomquist@utoledo.edu
Note: Load into slide show mode, and click mouse periodically to play animation show

## Slide 2
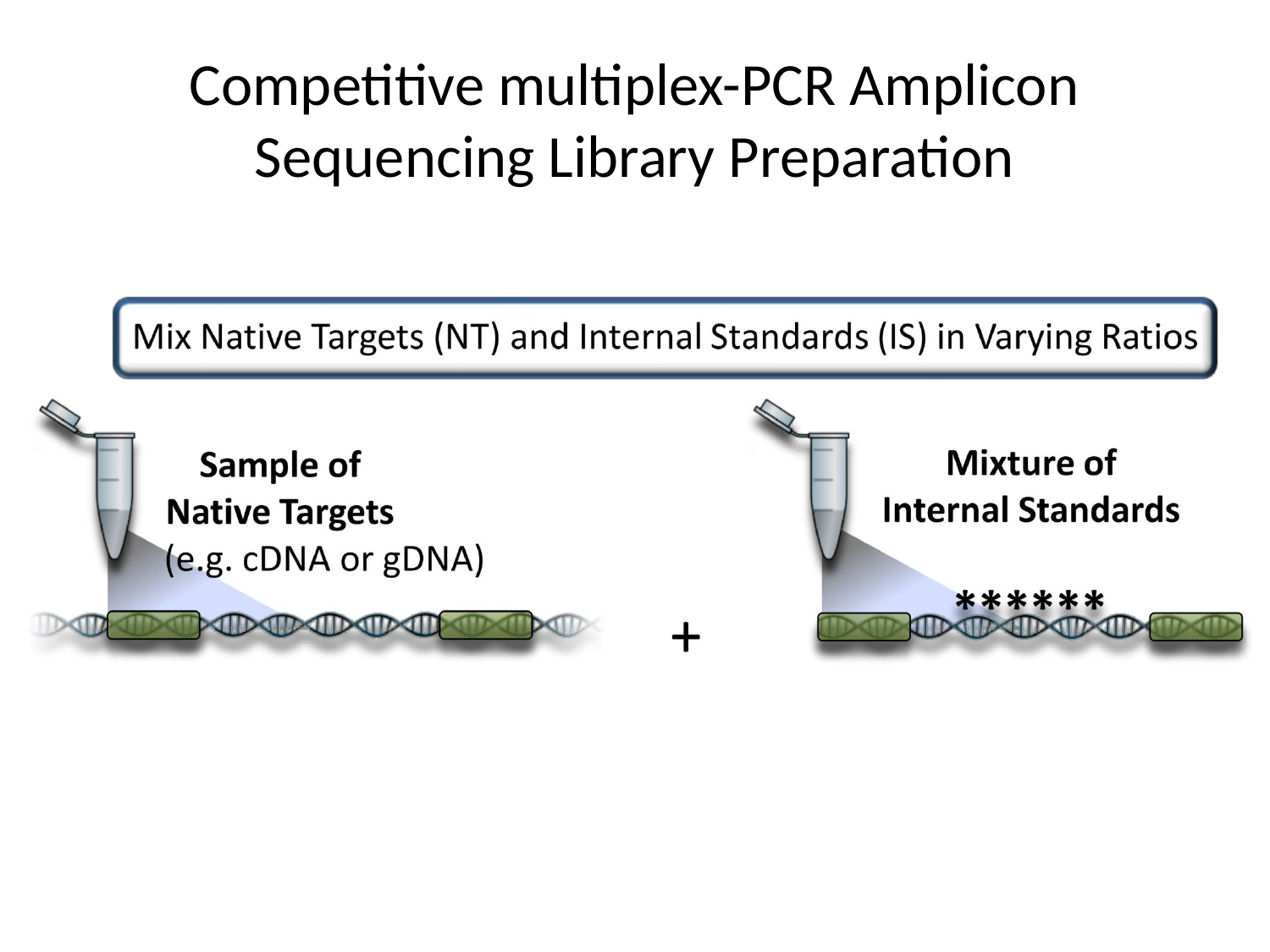

Competitive multiplex-PCR Amplicon Sequencing Library Preparation

## Slide 3
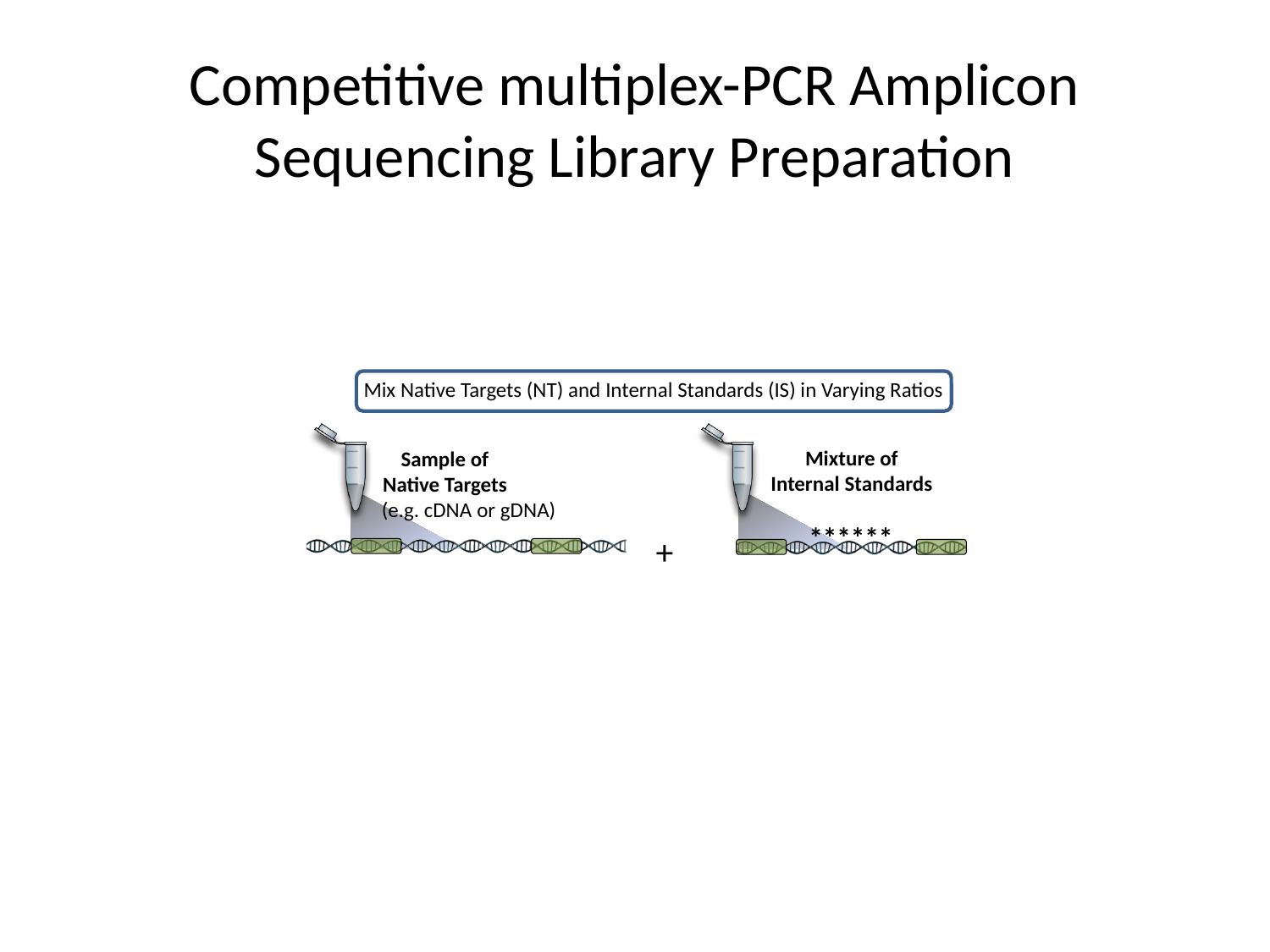

Competitive multiplex-PCR Amplicon Sequencing Library Preparation
Mix Native Targets (NT) and Internal Standards (IS) in Varying Ratios
Mixture of
Internal Standards
Sample of
Native Targets
(e.g. cDNA or gDNA)
******
+

## Slide 4
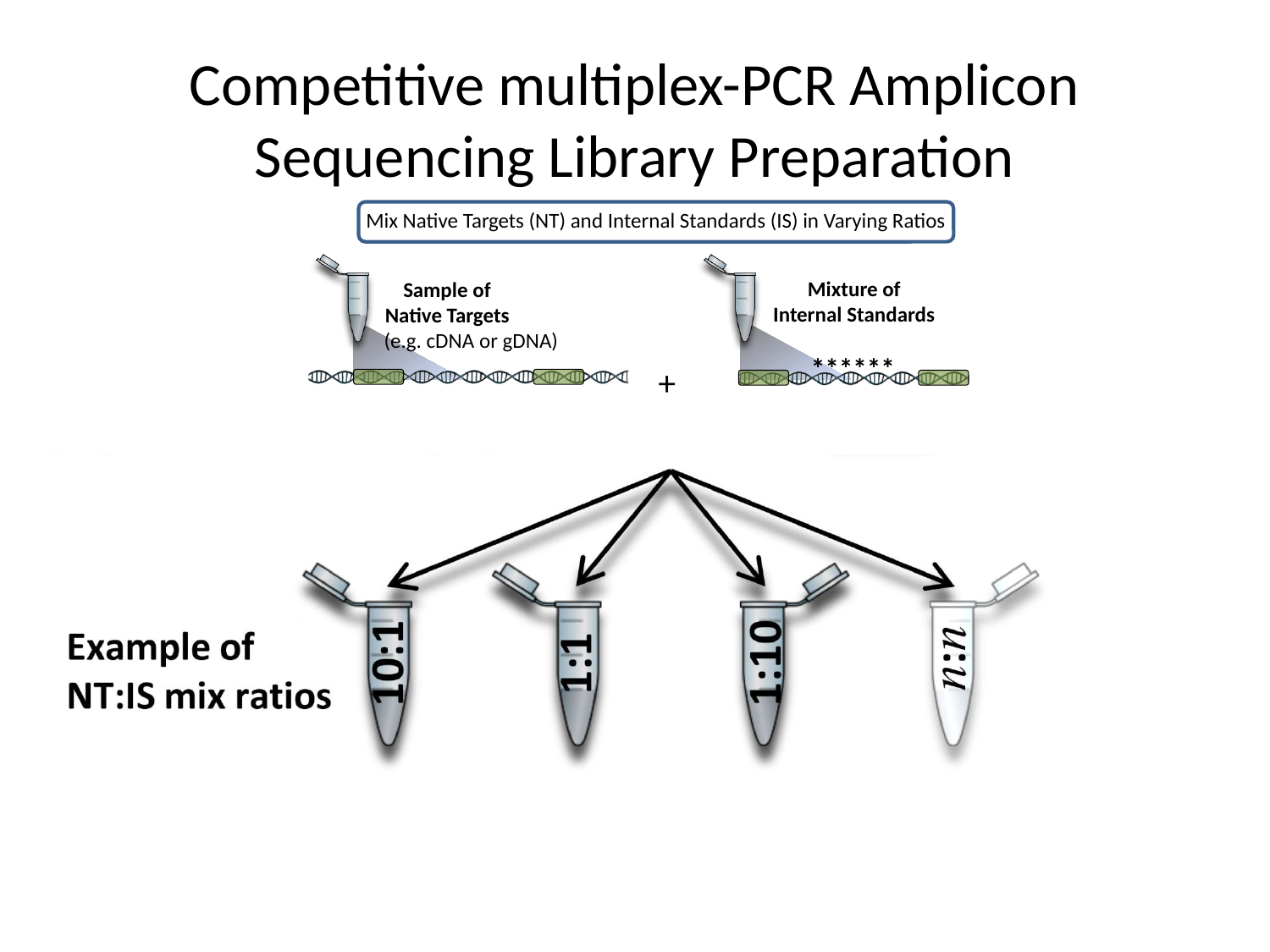

Competitive multiplex-PCR Amplicon Sequencing Library Preparation
Mix Native Targets (NT) and Internal Standards (IS) in Varying Ratios
Mixture of
Internal Standards
Sample of
Native Targets
(e.g. cDNA or gDNA)
******
+

## Slide 5
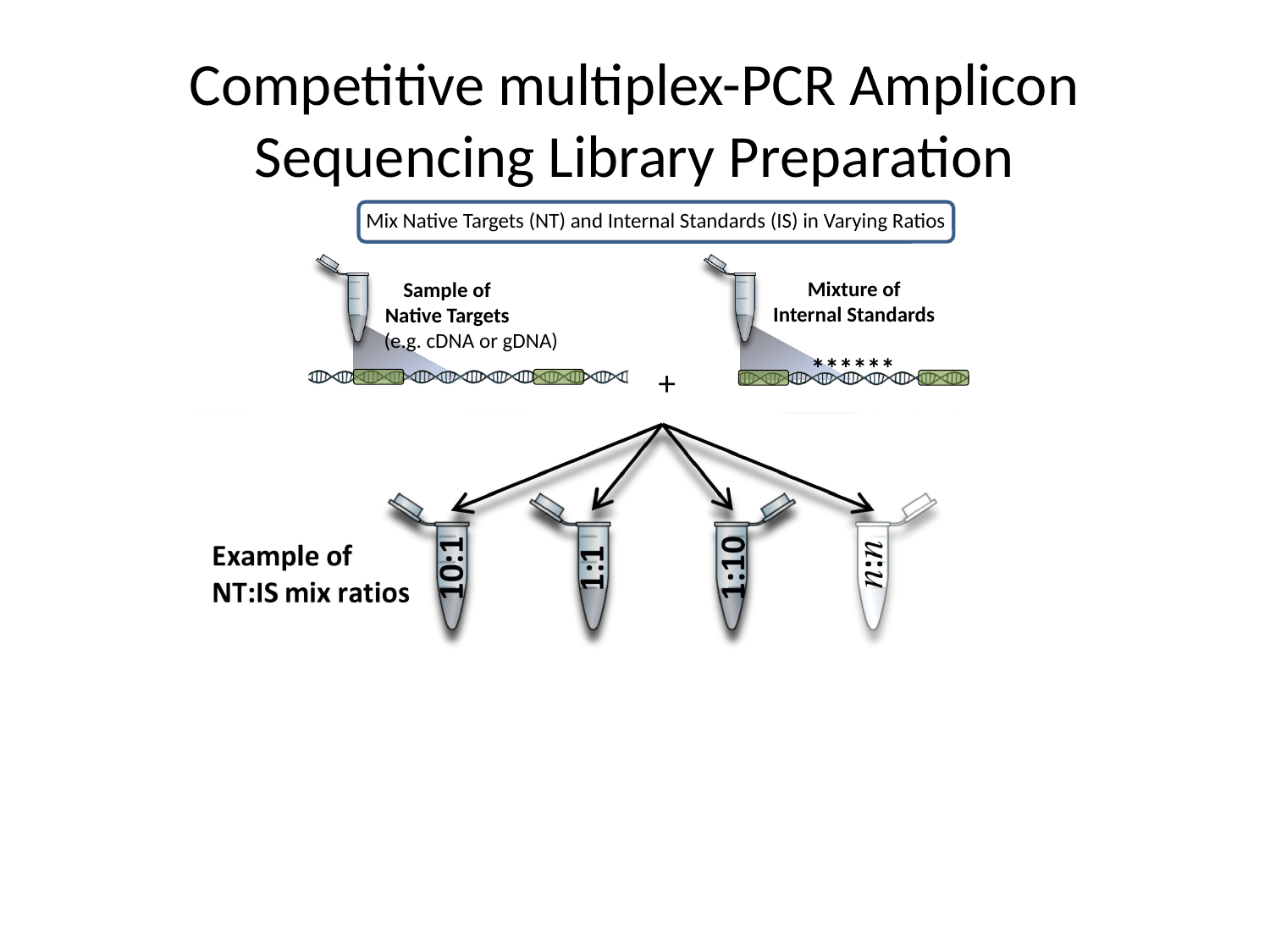

Competitive multiplex-PCR Amplicon Sequencing Library Preparation
Mix Native Targets (NT) and Internal Standards (IS) in Varying Ratios
Mixture of
Internal Standards
Sample of
Native Targets
(e.g. cDNA or gDNA)
******
+

## Slide 6
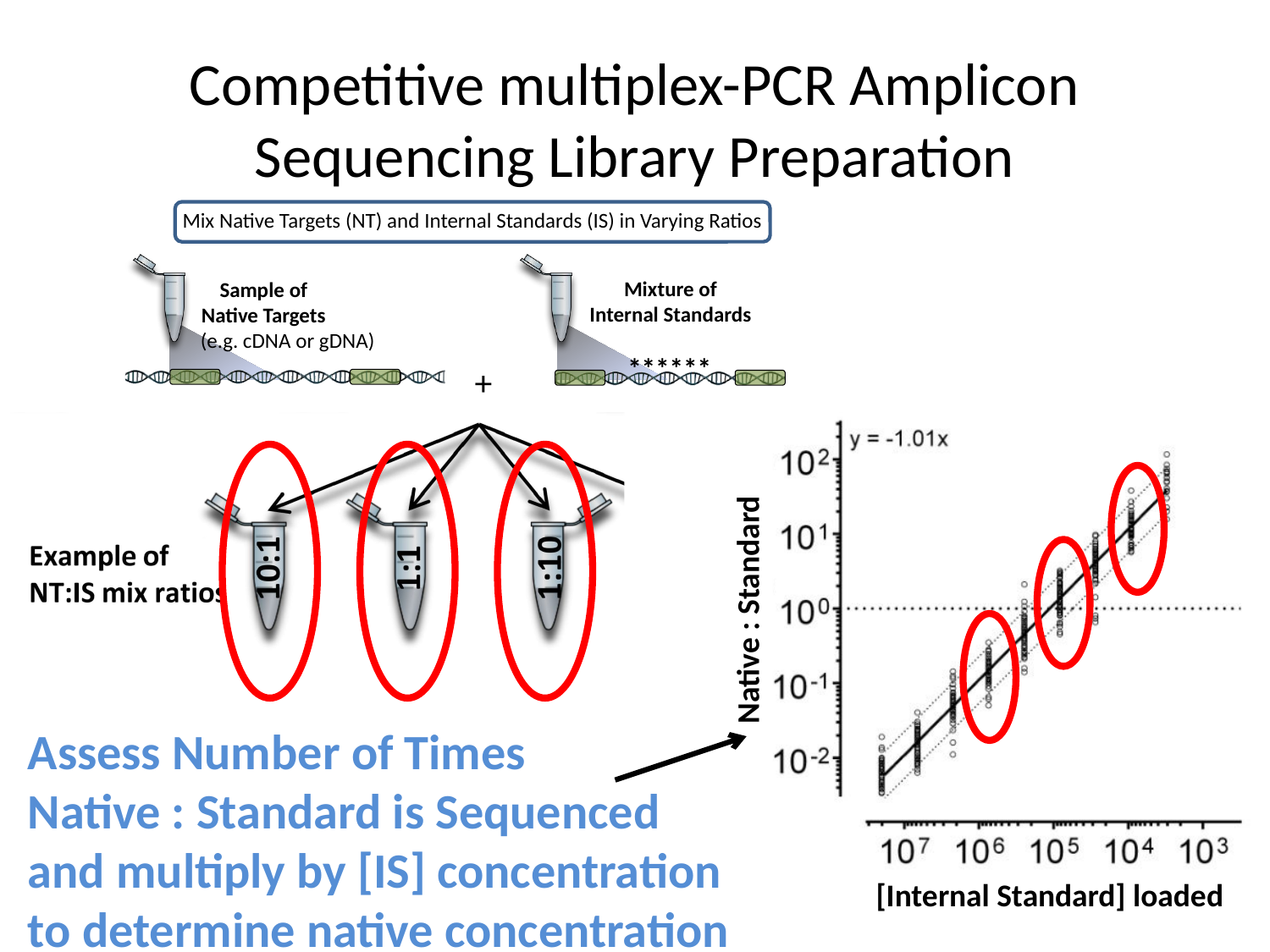

Competitive multiplex-PCR Amplicon Sequencing Library Preparation
Mix Native Targets (NT) and Internal Standards (IS) in Varying Ratios
Mixture of
Internal Standards
Sample of
Native Targets
(e.g. cDNA or gDNA)
******
+
Native : Standard
Assess Number of Times
Native : Standard is Sequenced
and multiply by [IS] concentration
to determine native concentration
[Internal Standard] loaded

## Slide 7
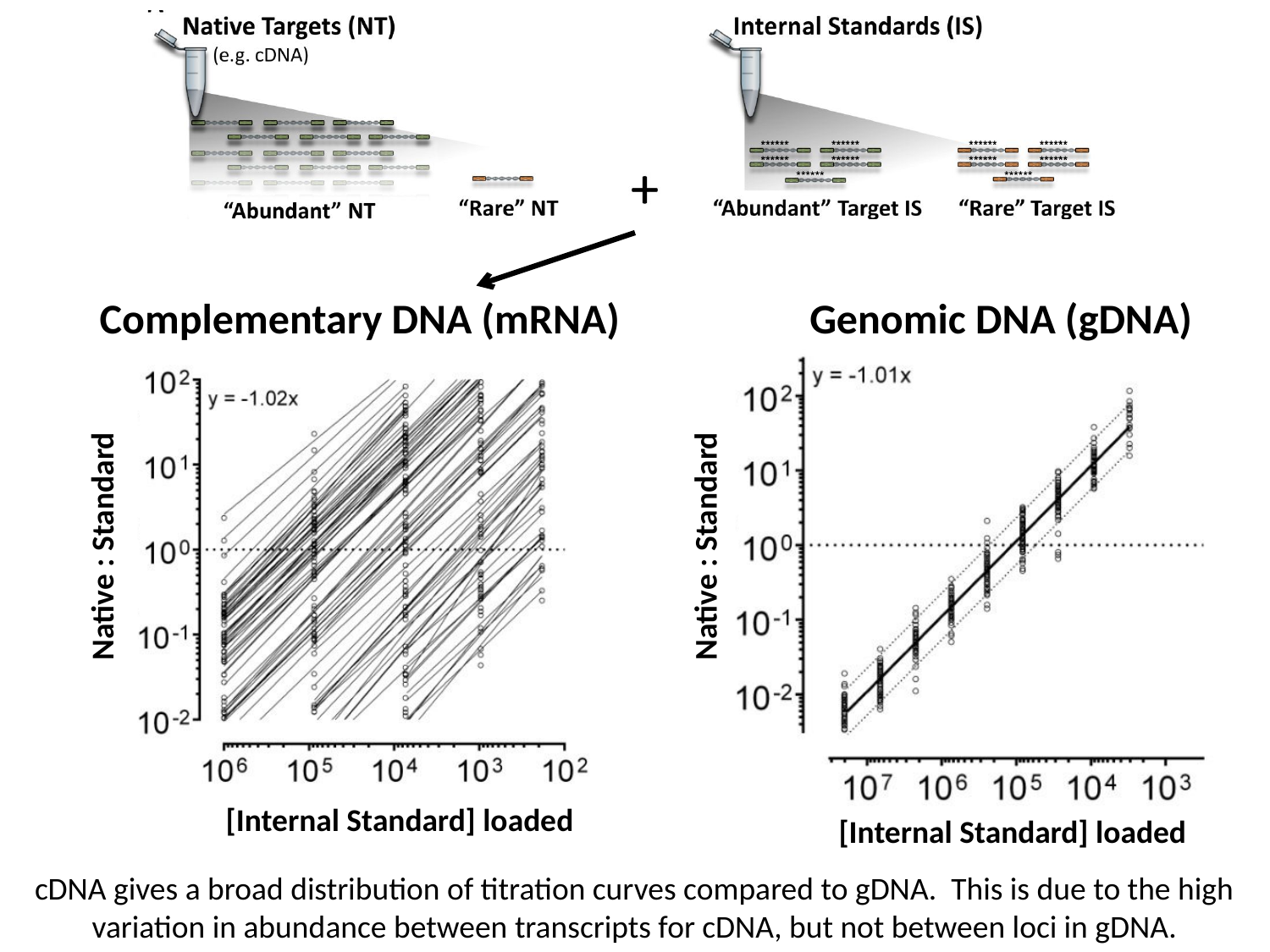

Complementary DNA (mRNA)
Genomic DNA (gDNA)
Native : Standard
Native : Standard
[Internal Standard] loaded
[Internal Standard] loaded
cDNA gives a broad distribution of titration curves compared to gDNA. This is due to the high variation in abundance between transcripts for cDNA, but not between loci in gDNA.

## Slide 8
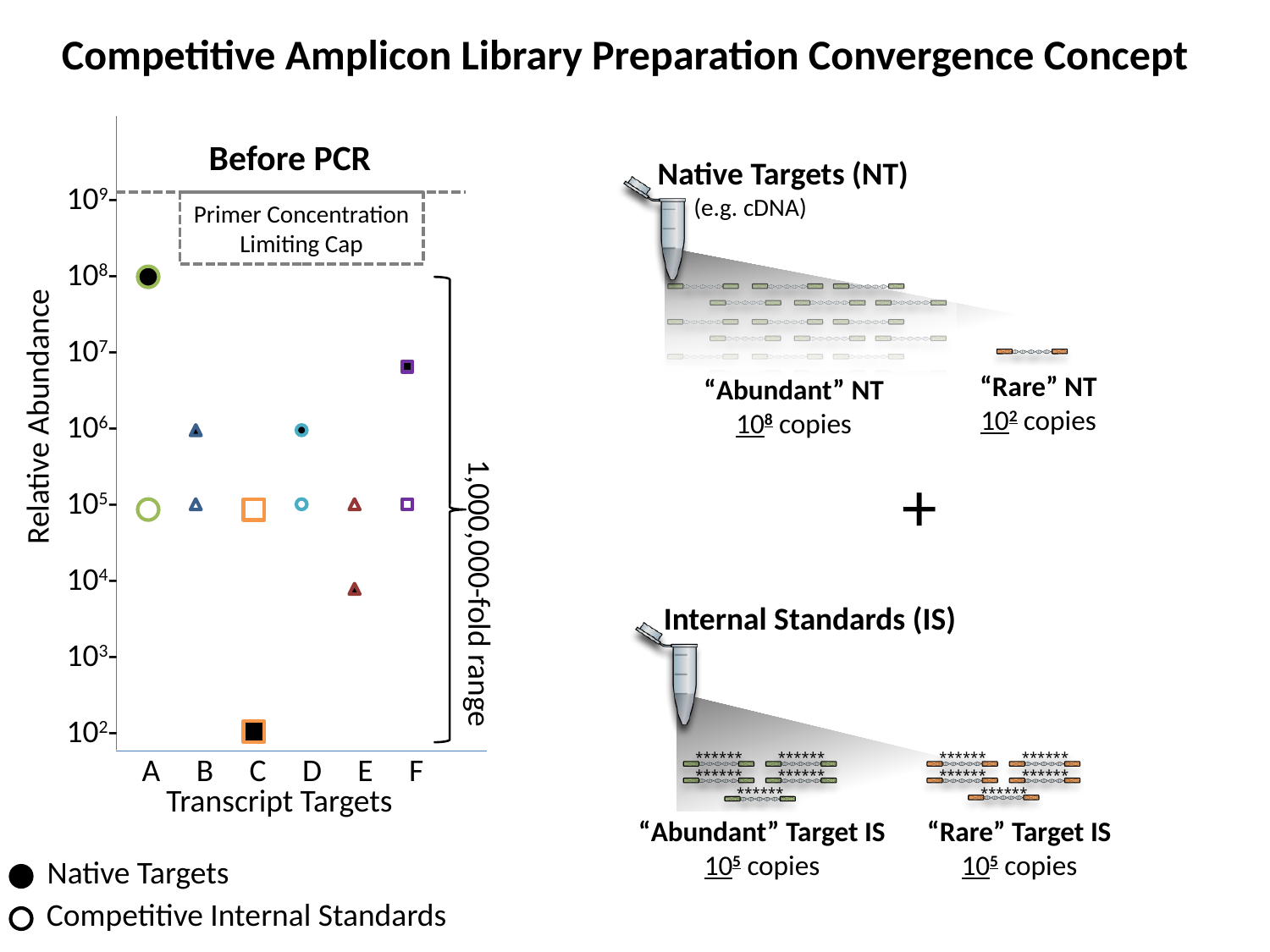

Competitive Amplicon Library Preparation Convergence Concept
109-
108-
107-
106-
105-
104-
103-
102-
Before PCR
Native Targets (NT)
 (e.g. cDNA)
Primer Concentration
Limiting Cap
“Rare” NT
102 copies
“Abundant” NT
108 copies
Relative Abundance
+
1,000,000-fold range
Internal Standards (IS)
******
******
******
******
******
******
******
******
******
******
A B C D E F
Transcript Targets
“Abundant” Target IS
105 copies
“Rare” Target IS
105 copies
Native Targets
Competitive Internal Standards

## Slide 9
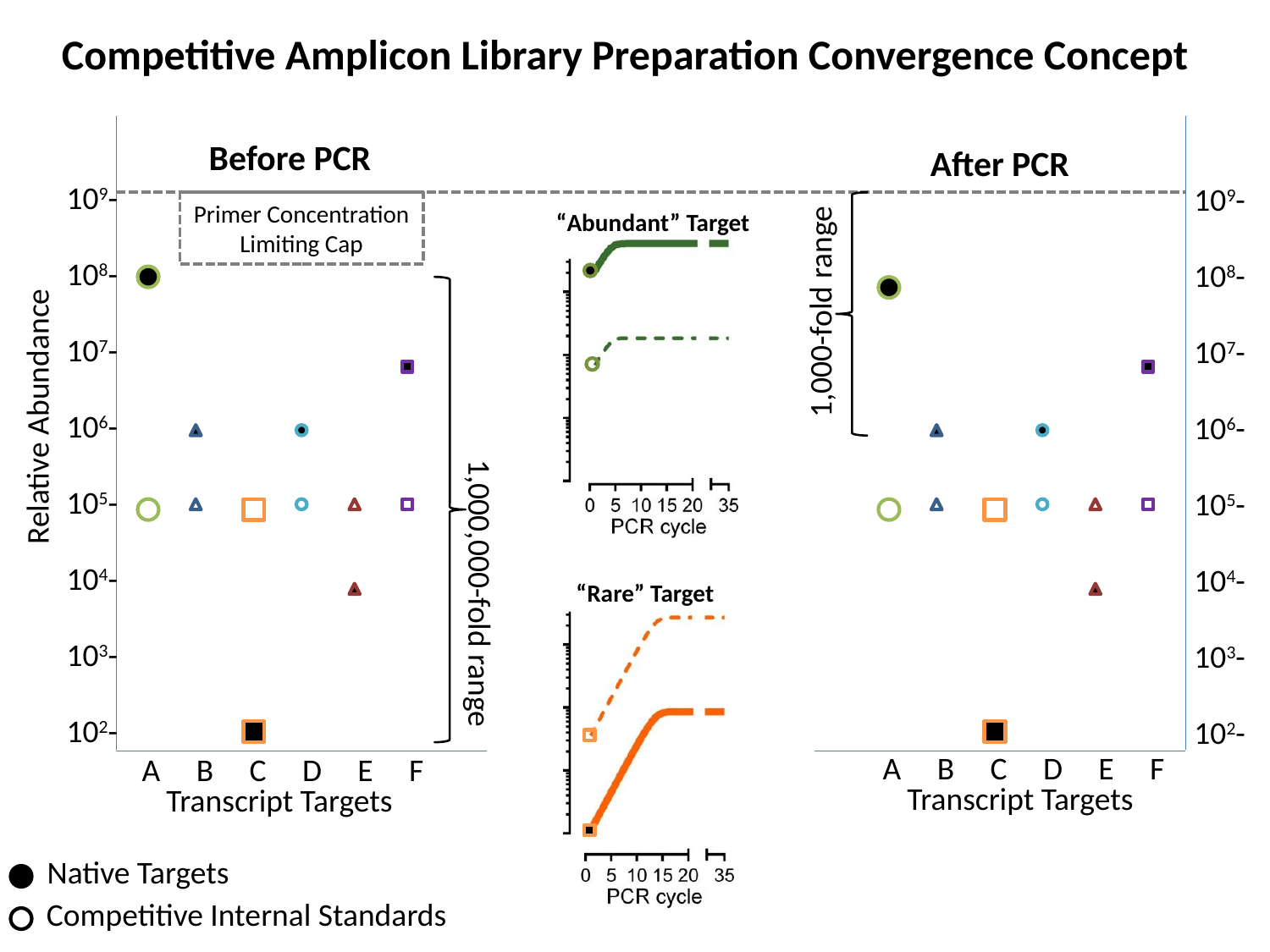

Competitive Amplicon Library Preparation Convergence Concept
109-
108-
107-
106-
105-
104-
103-
102-
109-
108-
107-
106-
105-
104-
103-
102-
Before PCR
After PCR
Primer Concentration
Limiting Cap
1,000-fold range
“Abundant” Target
Relative Abundance
1,000,000-fold range
“Rare” Target
A B C D E F
A B C D E F
Transcript Targets
Transcript Targets
Native Targets
Competitive Internal Standards

## Slide 10
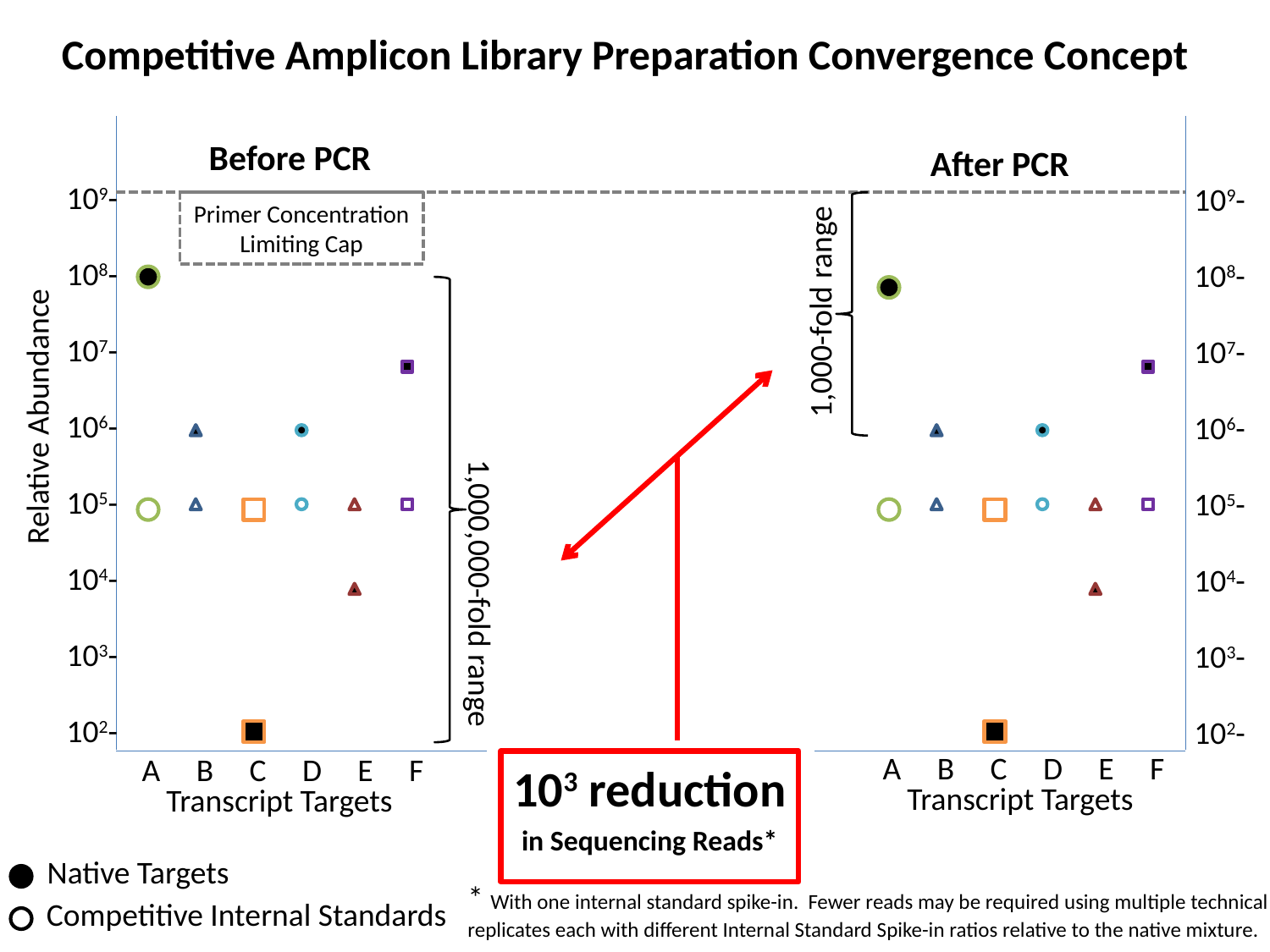

Competitive Amplicon Library Preparation Convergence Concept
109-
108-
107-
106-
105-
104-
103-
102-
109-
108-
107-
106-
105-
104-
103-
102-
Before PCR
After PCR
Primer Concentration
Limiting Cap
1,000-fold range
103 reduction
in Sequencing Reads*
Relative Abundance
1,000,000-fold range
A B C D E F
A B C D E F
Transcript Targets
Transcript Targets
Native Targets
* With one internal standard spike-in. Fewer reads may be required using multiple technical replicates each with different Internal Standard Spike-in ratios relative to the native mixture.
Competitive Internal Standards
